# Electroacupuncture promotes BDNF-dependent neurogenesis via microglial reprogramming in a chronic stress model

**DOI:** 10.1186/s13020-026-01334-y

**Published:** 2026-02-03

**Authors:** Lijuan Zhang, Ting Wei, Xuan Liu, Lifan Zhang, Dan Wang, Yucai Luo, Yanyan He, Zhaoxuan He, Fang Zeng

**Affiliations:** 1 Acupuncture and Tuina School, Chengdu University of Traditional Chinese Medicine, Chengdu, Sichuan, China; 2 Key Laboratory of Acupuncture for Senile Disease, Ministry of Education, Chengdu University of TCM, Chengdu, Sichuan China; 3 Acupuncture Point Effects Key Laboratory of Sichuan Province, Chengdu, Sichuan China; 4 Acupuncture Clinical Research Center of Sichuan Province, Chengdu, Sichuan China

**Keywords:** Electroacupuncture, Depression, Microglia, Neurogenesis, BDNF pathway

## Abstract

**Background:**

Aberrant microglial activation and impaired adult hippocampal neurogenesis play critical roles in the pathogenesis of depression. Although electroacupuncture (EA) has demonstrated clinical antidepressant efficacy, the underlying mechanisms by which it modulates microglial activity and promotes neurogenesis remain unclear.

**Methods:**

Male C57BL/6 J mice were subjected to chronic unpredictable mild stress (CUMS) for three weeks. Following this period, the mice were divided into groups receiving either EA at the Yintang (GV29) and Baihui (GV20) acupoints, imipramine (IMI) as a positive control, or no treatment (vehicle control) for an additional 3 weeks. To evaluate depressive-like behaviors, we conducted the sucrose preference test, forced swimming test, and tail suspension test. Anxiety-like behaviors were assessed using the open field test and elevated plus maze. We employed immunofluorescence, Golgi staining, Western blotting, and real-time quantitative PCR (qRT-PCR) to elucidate the effects of EA on microglia-driven hippocampal neurogenesis and BDNF signaling. Notably, loss-of-function experiments utilizing PLX5622 for microglial ablation and ANA-12 for TrkB blockade demonstrated the necessity of both microglia and BDNF signaling for the therapeutic efficacy of EA.

**Results:**

EA treatment significantly alleviated CUMS-induced anxiodepressive behaviors. This behavioral recovery was associated with a phenotypic shift in microglia towards a pro-neurogenic state in the hippocampus. Importantly, microglia were essential for the therapeutic effects of EA, as evidenced by their ablation with PLX5622. Furthermore, EA enhanced neurogenesis by orchestrating a multi-step augmentation of BDNF signaling, which involved PKA activation, subsequent release from MeCP2-mediated transcriptional repression, and ultimately increased maturation of BDNF.

**Conclusions:**

Our findings demonstrate that EA exerts antidepressant effects by promoting a pro-neurogenic transformation of microglia. Mechanistically, these microglia enhance BDNF function via the PKA/MeCP2/BDNF pathway, thereby facilitating hippocampal neurogenesis and restoring synaptic plasticity, which collectively alleviate depressive symptoms.

**Graphical Abstract:**

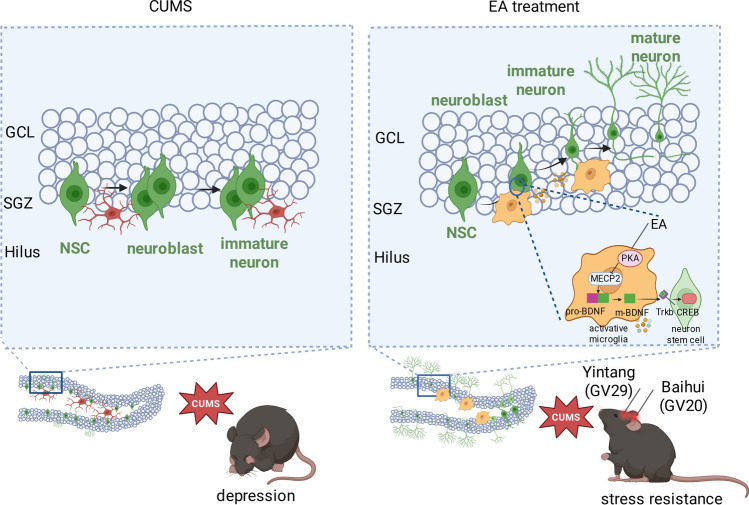

**Supplementary Information:**

The online version contains supplementary material available at 10.1186/s13020-026-01334-y.

## Introduction

Major depressive disorder (MDD) is a debilitating mental illness characterized by persistent low mood, anhedonia, cognitive impairment, and somatic symptoms. These symptoms often contribute to a heightened risk of suicidal ideation [1]. The etiology of MDD is complex, and chronic stressful life events are widely recognized as significant environmental risk factors [2]. To model this environmental influence, chronic unpredictable mild stress (CUMS) has been widely employed in rodent studies to reliably induce depressive-like behaviors [3]. Despite its prevalence, treatment remains challenging: current clinical antidepressants often yield poor prognoses, and novel drug candidates continue to exhibit high failure rates in clinical trials [4]. Given these limitations, there is a critical need to investigate the pathogenic mechanisms, which may help identify new therapeutic strategies.

The hippocampus plays a crucial role in mood regulation and cognitive function, with its dysfunction being a core pathophysiological feature of depression. A key mechanism underlying hippocampal plasticity is adult hippocampal neurogenesis (AHN). AHN involves the generation of new functional neurons from neural stem cells (NSCs) and their subsequent integration into the neural circuits of the dentate gyrus (DG) [5]. This process is essential for maintaining the structural and functional integrity of the hippocampus and significantly contributes to mood regulation [6]. CUMS induces profound hippocampal pathology, including volume reduction, decreased cell proliferation, and impaired neuronal differentiation [7]. Notably, multiple antidepressants, including selective serotonin reuptake inhibitors, exert their therapeutic effects, at least partially, by enhancing AHN in the DG of adult rodents [8, 9]. Microglia play a dual role in regulating AHN, under physiological conditions, they support neurogenesis through the release of neurotrophic factors and phagocytic clearance of debris [10]. However, under pathological conditions such as chronic stress, activated microglia impair the maintenance of NSCs and hinder the maturation of new neurons by releasing inflammatory factors [11]. Similar to addiction, the microglial inhibitor minocycline reverses the pathogenic phagocytic activity of neurotoxic microglia and ameliorates the deficits in neurogenesis induced by chronic stress [12]. Microglia critically rely on colony-stimulating factor 1 receptor (CSF1R) signaling for their survival [13]. Depletion of microglia using the CSF-1R antagonist PLX5622 completely abrogated the therapeutic effects on depressive-like behavior and neurogenesis impairment [14]. Furthermore, we have previously demonstrated that IL4-mediated microglia enhance AHN and ameliorate depressive-like behaviors in CUMS mice [15]. However, direct cytokine-based interventions (e.g., intracerebral delivery) are often invasive, while systemic pharmacological approaches face translational challenges such as off-target effects and limited blood–brain barrier penetration. Therefore, there is a compelling need to identify non-invasive alternative strategies that can effectively modulate microglial function and promote neurogenesis.

Acupuncture, particularly electroacupuncture (EA), has garnered attention as a potential non-pharmacological intervention for alleviating depressive symptoms with minimal side effects [16]. Studies have showed that acupuncture interventions yield positive outcomes for depression, anxiety, quality of life, and symptom severity in patients [17]. Preclinical research further indicates that EA mitigates depressive-like behaviors in rodent models by reducing neuroinflammation and activating the brain-derived neurotrophic factor (BDNF) pathway. Together, these actions work to restore neural plasticity [12, 18]. Nevertheless, the precise molecular mechanisms through which EA regulates BDNF to improve depressive behavior remain unclear.

As a central mediator of neuroplasticity, BDNF plays a crucial role in facilitating hippocampal neurogenesis. Clinically, serum BDNF levels are consistently lower in individuals with MDD compared to healthy controls [19], and peripheral BDNF levels often exhibit an inverse correlation with the severity of depression [20]. Growing evidence from rodent models indicates that increasing hippocampal BDNF levels induces long-lasting antidepressant-like effects [21, 22]. The expression of BDNF is transcriptionally regulated by Methyl-CpG Binding Protein 2 (*MeCP2*). The mutations in MeCP2 directly associated with severe neuronal impairment and motor deficits [23, 24]. BDNF is synthesized as a precursor protein, pro-BDNF, which undergoes proteolytic cleavage to yield the mature, biologically active form (mature BDNF). Several studies have demonstrated that pro-BDNF and mature BDNF exhibit opposing biological effects on neuronal survival, plasticity, and synaptic function [20]. Consequently, the pro-BDNF/BDNF ratio appears to be disrupted and plays a significant role during antidepressant treatments [25]. Furthermore, the Protein Kinase A (PKA) signaling pathway serves as another critical regulator of BDNF expression and function. Its activation strongly implicated in the pathophysiology and treatment of depression [26].

BDNF exerts its effects primarily through binding to its cognate receptor, tropomyosin receptor kinase B (TrkB). This interaction regulates key brain functions including neuronal survival, neurogenesis, and neuroplasticity [27]. Importantly, deficits in the BDNF-TrkB axis are evident in depression. Postmortem studies have demonstrated the decreased TrkB mRNA levels in depressed patients, and genetic variants in the TrkB gene are associated with an increased risk of suicide [28, 29]. Upon TrkB activation, BDNF signaling promotes the phosphorylation of the cAMP response element-binding (CREB) protein. Phosphorylated CREB then stimulates the transcription of genes essential for synaptic plasticity and hippocampal neurogenesis [30]. Notably, modulating microglial activity—such as shifting them toward a neuroprotective phenotype—can restore synaptic and neuronal function in depression models. This recovery is partly mediated through upregulation of the BDNF-TrkB-CREB pathway [31, 32]. Thus, beyond neuronal mechanisms, microglia serve as a critical regulatory node that actively supports BDNF-dependent functional recovery. However, the integration of these distinct pathways within microglia to promote neurogenesis remains to be fully elucidated.

In this study, we hypothesized that EA exerts its antidepressant effects by driving a pro-regenerative phenotype in microglia, which in turn orchestrates the concurrent activation of these multiple BDNF-induction pathways. To test this, we employed a combined strategy of microglial depletion (using PLX5622) and BDNF-TrkB signaling blockade (using ANA-12). Our results demonstrate a causal role for microglia in integrating this BDNF response and in mediating the behavioral benefits of EA.

## Results

### EA treatment ameliorates CUMS-induced depressive-like behaviors

**Fig. 1:**
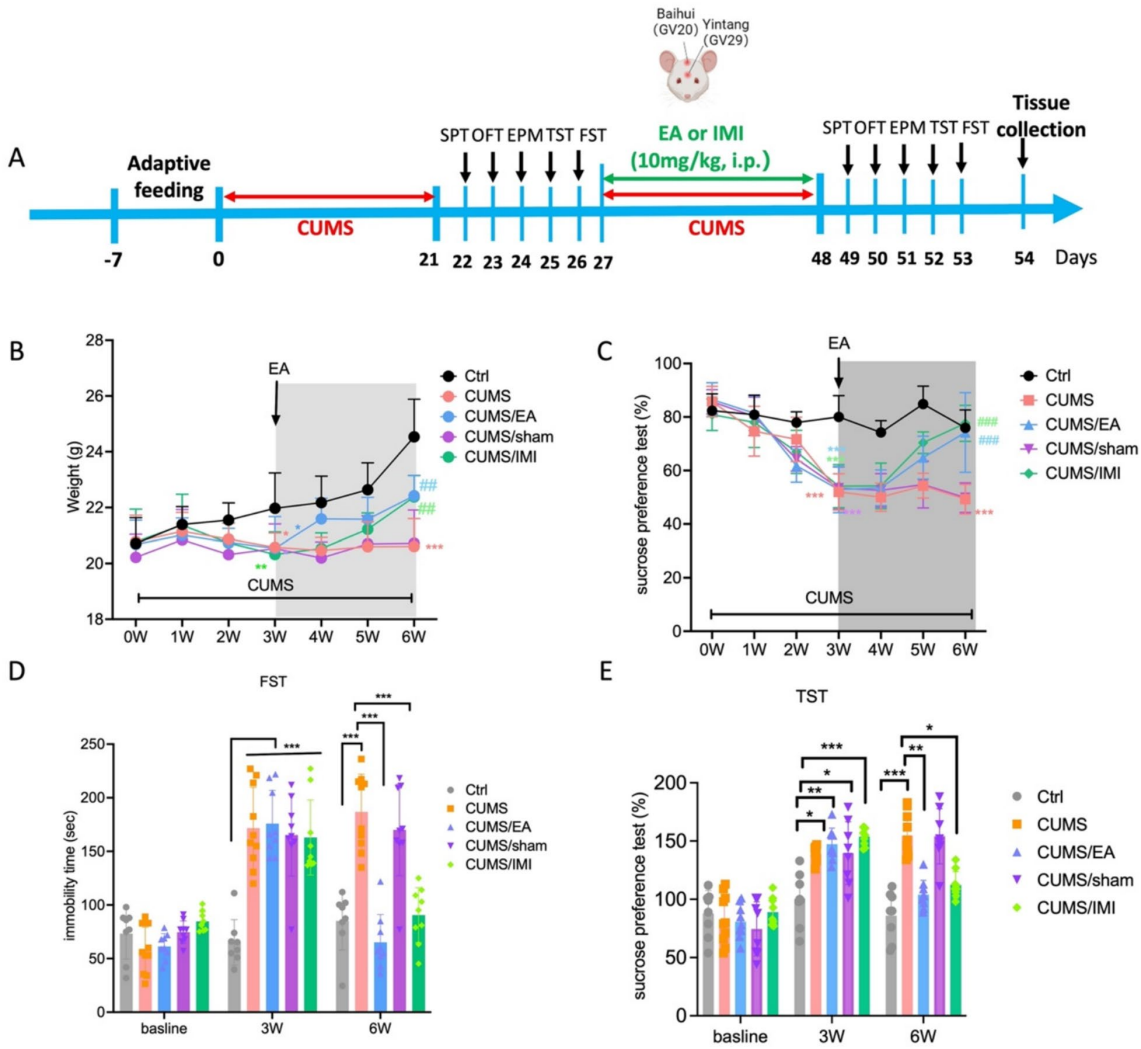
EA treatment alleviated the CUMS-induced depressive-like behaviors. **A** Experimental timeline of the CUMS paradigm and subsequent treatments. **B** Effect of EA on the changes of body weight of CUMS-exposed mice. **C** Effect of EA on the changes of sucrose preference index of CUMS-exposed mice. Mice treated by EA and imipramine were assessed the immobility time in FST (**D**) and TST (**E**) at baseline, 3 weeks, and 6 weeks after CUMS procedure. CUMS, chronic unpredictable mild stress; IMI, imipramine; EA, electroacupuncture; SPT, sucrose preference test; FST, force swimming test; TST, tail suspension test. Data are presented as mean ± SEM (n = 8 for Ctrl and CUMS/sham groups; n = 9 for CUMS/EA and CUMS/IMI groups; n = 10 for CUMS group). *p < 0.05, **p < 0.01, ***p < 0.001 vs. Ctrl group; #p < 0.05, ##p < 0.01, ###p < 0.001 vs. CUMS group (two-way ANOVA followed by Tukey’s multiple comparisons test). For detailed F-values, degrees of freedom, and exact p-values of all comparisons, see Supplementary Table S1

To investigate the effect of EA on CUMS-induced behavioral disorders, mice were subjected to CUMS for three weeks followed by a three-week treatment period with EA, imipramine (IMI), or sham EA (Fig. 1A). A battery of behavioral tests, including the sucrose preference test (SPT), open field test (OFT), elevated plus maze (EPM), forced swim test (FST), and tail suspension test (TST), were conducted in an order of increasing stress intensity, with a 24-h rest period between tests to minimize carry-over effects [33, 34]. A two-way repeated-measures ANOVA on body weight indicated a significant main effect of time (F = 21.84, p < 0.001), a significant main effect of treatment (F = 14.34, p < 0.001), and a significant time × treatment interaction (F = 5.335, p < 0.001). Post hoc analysis confirmed that CUMS exposure significantly attenuated weight gain in mice compared to the control group (p < 0.001). Both EA and IMI treatment effectively restored normal weight gain relative to the CUMS group (p < 0.01, Fig. 1B). Similarly, analysis of sucrose preference revealed a significant time × treatment interaction (F = 11.33, p < 0.001). Post hoc analysis confirmed that after 3 weeks of CUMS, sucrose intake was significantly reduced in the CUMS group compared to controls (p < 0.001). After three weeks of treatment, both EA and IMI significantly increased sucrose intake compared to the CUMS group (p < 0.001 for both comparisons) (Fig. 1C). Additionally, a two-way repeated-measures ANOVA of immobility times revealed a significant interaction between time and treatment in both the TST (F = 18.21, p < 0.001) and the FST (F = 7.031, p < 0.001). Post hoc analysis confirmed that CUMS exposure significantly increased immobility times at the three-week time point (p < 0.001 vs. control). Subsequent EA and IMI treatment significantly reversed these effects, reducing immobility times at the 6-week time point compared to the CUMS group (Fig. 1D–E). In contrast, sham EA treatment failed to significantly reverse any of the CUMS-induced behavioral deficits.

### EA treatment diminishes anxiety-like behaviors in CUMS-exposed mice

**Fig. 2:**
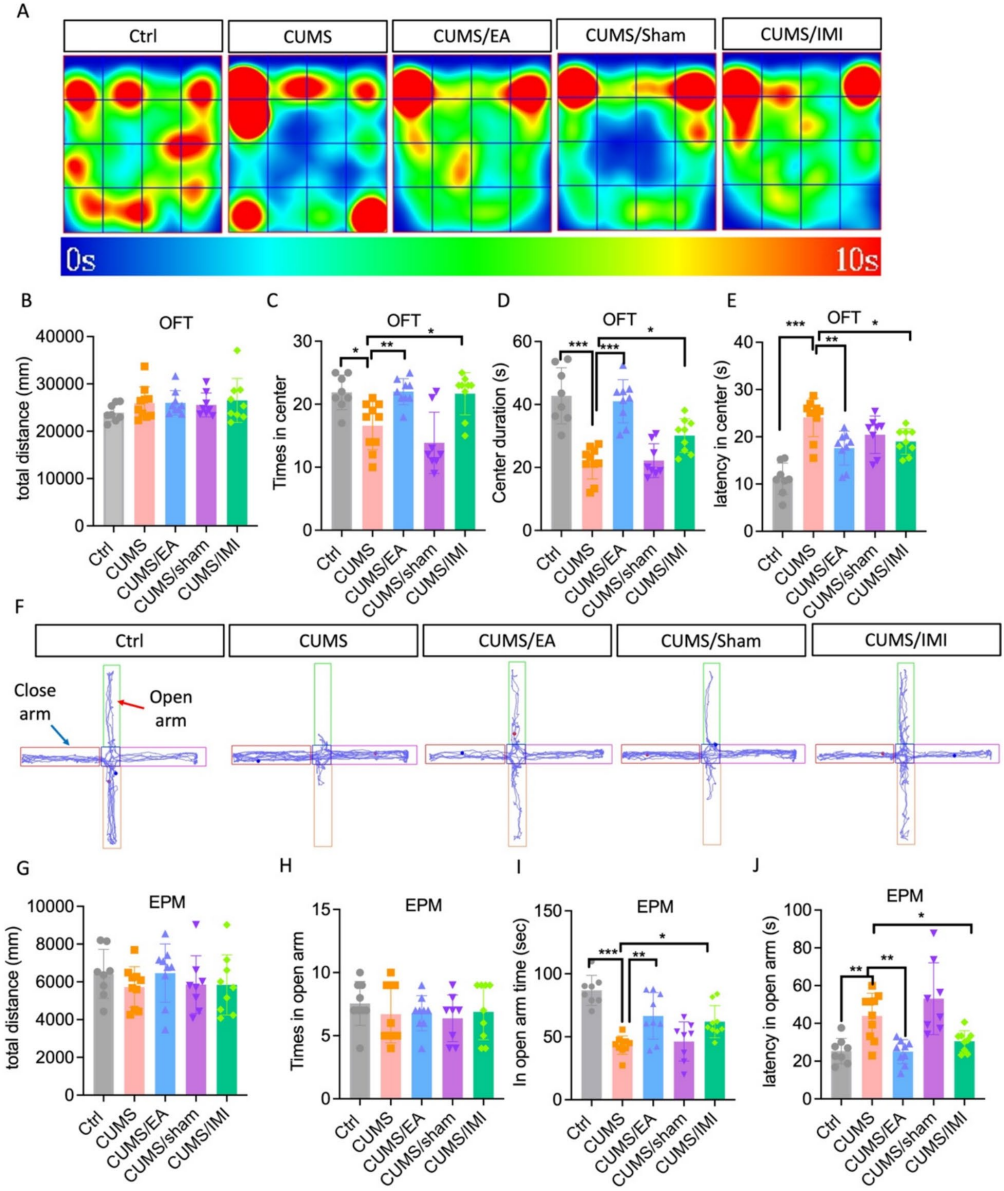
EA treatment reverses CUMS-induced anxiety-like behaviors. **A** Heatmap of the trajectory of mice in OFT after EA-treated in CUMS exposure. Total distance traveled in the OFT (**B**), the number of entries in the center of OFT (**C**), time spent in center of the open field (**D**), and latency in the center (**E**) after EA-treated in CUMS exposure. **F** Representative tracks of mice in EPM after EA-treated in CUMS exposure. **G** Total traveled distance during the EPM. **H** The number of entries in the open arms. **I** Time spent and **J** latency in the open arms of the EPM. CUMS versus control, and EA or sham versus CUMS. OFT, open field test; EPM, elevated plus maze. Data are presented as mean ± SEM (n = 8 mice for Ctrl and CUMS/sham groups, n = 9 mice for CUMS/EA and CUMS/IMI groups, n = 10 mice for CUMS group). *p < 0.05, **p < 0.01, ***p < 0.001. Statistical analysis was performed by one-way ANOVA followed by Tukey’s post hoc test. For detailed F-values, degrees of freedom, and exact p-values of all comparisons, see Supplementary Table S2

The anxiolytic effects of EA and IMI were evaluated using the open field test (OFT) and the elevated plus maze (EPM) in mice subjected to CUMS. In the OFT, no significant differences in total distance traveled were observed among groups (Fig. 2A–B), indicating that locomotor activity remained unaffected. A one-way ANOVA revealed a significant treatment effect on the time spent in the center (F = 9.850, p < 0.001) and the number of entries into the center zone (F = 21.44, p < 0.001). Post hoc analysis confirmed that compared to the control group, CUMS-exposed mice spent significantly less time in the center and made fewer entries into the zone compared to control. Both EA and IMI treatment effectively normalized these measures, significantly increasing both center time and entries relative to the CUMS group (Fig. 2C–D). Similarly, center entry latency was significantly influenced by treatment (F = 15.42, p < 0.001), with CUMS mice exhibiting prolonged latency compared to control (p < 0.001). This effect was significantly mitigated by both EA and IMI treatments (Fig. 2E). Sham EA did not reverse any CUMS-induced alterations in OFT parameters (Fig. 2C–E). In the EPM, no group differences were found in total distance traveled or open-arm entries (Fig. 2G–H), further confirming comparable locomotor activity. One-way ANOVA identified a significant treatment effect on open-arm exploration time (F = 14.21, p < 0.001). Post hoc tests confirmed that CUMS significantly reduced open-arm time compared to control (p < 0.001), an effect that was reversed by both EA and IMI treatments (Fig. 2I). Likewise, treatment significantly affected the latency to first enter an open arm (F = 10.79, p < 0.001). CUMS prolonged this latency relative to control (p < 0.01), and EA and IMI treatments significantly shortened it compared to the CUMS group (Fig. 2J). Sham EA produced no significant improvements in EPM behaviors (Fig. 2I–J).

### EA treatment enhance adult hippocampal neurogenesis in CUMS-exposed mice

**Fig. 3:**
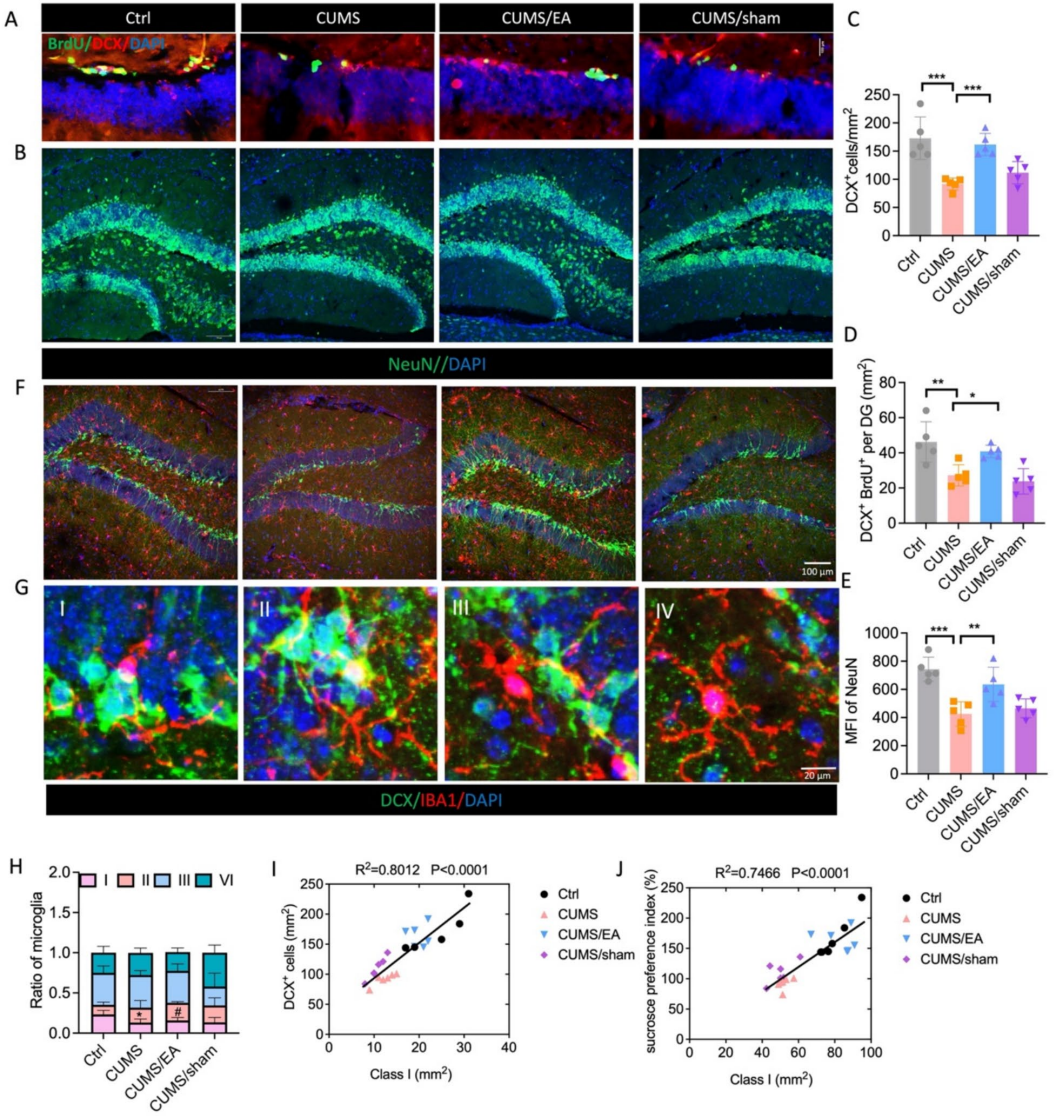
EA enhanced hippocampus neurogenesis in CUMS mice. **A** Representative confocal images of DCX^+^ and BrdU^+^ cells in DG regions of the ventral hippocampus. Scale bars, 100 μm. **B** Representative images of NeuN^+^ cells in hippocampus. **C** Quantification of the number of DCX^+^ in DG area. n = 5 mice. One-way ANOVA with Tukey post hoc test was performed for statistical analysis. **D** The number of DCX^+^ BrdU^+^ cells in hippocampus. n = 5 mice. One-way ANOVA with Tukey post hoc test was performed for statistical analysis. **E** MFI of NeuN in DG area of EA-treated mice. n = 5 mice. One-way ANOVA with Tukey post hoc test was performed for statistical analysis. **F** Representative confocal images of DCX^+^, and IBA1^+^ cells in DG regions of the hippocampus. Scale bars, 100 μm. **G** Quantitative analysis of microglia-neuron interactions. 1, microglia “embracing” neurons; II, microglial cell bodies in close contact with neuron bodies without embracing; III, microglial branches in close contact with neuron bodies without evidence of intercellular contact; IV, no microglia in clear contact with neuron bodies seen in the images. Scale bars, 20 μm. **H** Quantification of microglia-neuron interactions in EA-treated mice. n = 5 mice. Two-way ANOVA followed with Tukey’s multiple comparisons test. **I** Correlation between the proportion of microglia exhibiting Class I morphology and the number of DCX^+^ cells in DG. n = 5 mice. Data were analyzed by Pearson’s correlation. **J** Correlation between the proportion of microglia exhibiting Class I morphology and sucrose preference. n = 5 mice. Data were analyzed by Pearson’s correlation. The values are presented as mean ± SEM. *p < 0.05, **p < 0.01, ***p < 0.001 versus control (Ctrl) group, #p < 0.05, ##p < 0.01, ###p < 0.001 versus CUMS group. MFI, mean fluorescence intensity. For detailed F-values, degrees of freedom, and exact p-values of all comparisons, see Supplementary Table S3

Emerging evidence indicates that hippocampal neurogenesis enhances stress resilience [15]. Thus, we investigated whether the antidepressant effect of EA is associated with the promotion of adult hippocampal neurogenesis. Neurogenesis is a multi-step process involving progenitor proliferation, neuronal differentiation, and maturation. Immunofluorescence was employed to label DCX^+^ cells (immature neurons), BrdU^+^/DCX^+^ cells (proliferating neuronal progenitors), and NeuN^+^ cells (mature neurons) (Fig. 3A–B). A one-way ANOVA revealed a significant effect of treatment on the number of DCX⁺ cells (F = 12.99, p < 0.001). Post hoc analysis confirmed that CUMS significantly decreased the number of DCX⁺ cells compared to controls (p < 0.001). Importantly, EA treatment effectively prevented this reduction, resulting in significantly higher counts than the CUMS group (p < 0.001) (Fig. 3C). Similarly, a one-way ANOVA on BrdU⁺/DCX⁺ cells demonstrated a significant treatment effect (F = 9.777, p < 0.001). CUMS exposure significantly reduced the number of proliferating neuronal progenitors (p < 0.01 vs. control), while EA treatment significantly increased their number compared to the CUMS group (p < 0.05) (Fig. 3D). Furthermore, CUMS significantly reduced the mean fluorescence intensity (MFI) of NeuN, suggesting compromised neuronal maturity or density. In contrast, EA treatment significantly increased the MFI of NeuN compared to the CUMS group (p < 0.01) (Fig. 3E). To investigate microglia-mediated neurogenesis in the DG, we assessed physical interactions between microglia (IBA1^+^) and immature neurons (DCX^+^) using immunofluorescence (Fig. 3F). Interactions were classified based on proximity and morphology (Fig. 3G): microglia in close contact with neuronal bodies by processes that wrap around the neuron (Class I) or not (Class II), microglia processes in close contact with neuronal bodies (Class III), and microglia not in close contact with neuronal bodies (Class IV). A two-way repeated-measures ANOVA with Treatment Group and DCX Type as factors revealed a significant main effect of DCX Type (F = 27.29, p < 0.0001) and a significant Treatment × DCX Type interaction (F = 3.560, p = 0.0030). Post-hoc analysis using Tukey’s test indicated that CUMS exposure significantly reduced the proportion of intimate Class I interactions compared to controls (p < 0.05). Critically, EA treatment significantly increased the proportion of Class I interactions relative to the CUMS group (Fig. 3H). To functionally link this specific microglial morphological shift with the observed neurogenic and behavioral outcomes, we performed Pearson correlation analyses. The result showed that the proportion of Class I microglia was significantly positively correlated with the number of DCX⁺ cells in the DG (r = 0.8012, n = 24, p < 0.001; Fig. 3I), and was also significantly positively correlated with sucrose preference (r = 0.7466, n = 24, p < 0.001; Fig. 3J). Collectively, these results demonstrate that EA treatment promotes adult hippocampal neurogenesis in CUMS-exposed mice.

### EA reprograms microglia toward a pro-neurogenic phenotype in the hippocampus of CUMS mice

**Fig. 4:**
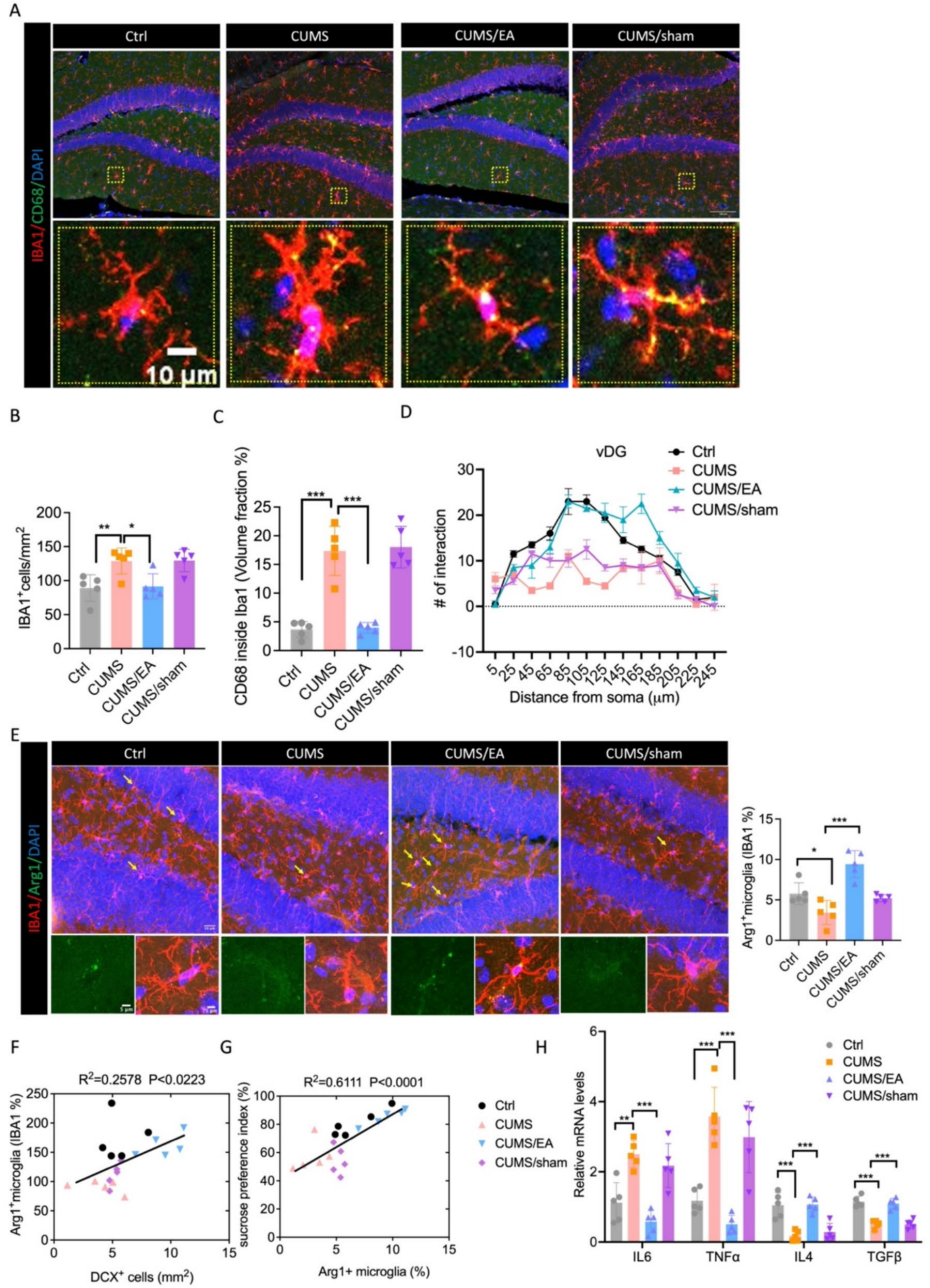
EA induced pro-neurogenesis microglia in the hippocampus of CUMS mice. **A** Representative images of IBA1 (red) and CD68 (green) immunostaining in the hippocampus. Scale bars = 100 μm. Zoom in images (Scale bars = 10 μm). **B** The immunostaining quantitative analysis of number of cells positively labeled for IBA1. n = 5 mice. One-way ANOVA followed by Tukey’s post hoc test. **C** Quantitative analyses of colocalization of CD68 in IBA1^+^cells. n = 5 mice. One-way ANOVA followed by Tukey’s post hoc test. **D** Sholl analysis of hippocampal microglia. Two-way repeated-measures ANOVA with Tukey’s post hoc test. n = 5 mice. **E** Representative images of IBA1 (red) and Arg1 (green) immunostaining in the hippocampus, and quantification of hippocampal Arg1^+^ microglia. Scale bars = 20 μm. Zoom in images (Scale bars = 5 μm). **F** Correlation between the number of Arg1^+^ microglia and DCX^+^ cells. n = 5 mice. Data were analyzed by Pearson’s correlation. **G** Correlation between the number of Arg1^+^ microglia and sucrose preference index. n = 5 mice. Data were analyzed by Pearson’s correlation. **H** The concentration of IL6, TNFα, IL4, TGFβ mRNA level in hippocampus, n = 5 mice. One-way ANOVA followed by Tukey’s post hoc test. The values are presented as mean ± SEM. *p < 0.05; **p < 0.01; ***p < 0.001. For detailed F-values, degrees of freedom, and exact p-values of all comparisons, see Supplementary Table S4

A growing body of research suggested that depression was often accompanied by microglial overactivation [35]. Given that microglia play a critical role in shaping the neurogenic niche, we hypothesized that EA alleviates depressive-like behaviors by reprogramming microglia. We first assessed general microglial activation. One-way ANOVA revealed that CUMS exposure increased microglial density (IBA1 cells, p < 0.01) and phagocytic activity (CD68 area, p < 0.001) in the DG, both of which were normalized by EA treatment (Fig. 4A–C). Concomitantly, EA prevented the CUMS-induced reduction in microglial process complexity (Sholl analysis, Fig. 4D), indicating a restoration of homeostatic surveillance. To specifically test for a pro-neurogenic phenotype, we quantified Arg1^+^ microglia. EA significantly increased the proportion of Arg1^+^ microglia in the DG of CUMS-exposed mice (Fig. 4E, p < 0.001). Crucially, Pearson’s correlation analysis demonstrated that the abundance of these Arg1^+^ microglia was positively correlated with the number of DCX^+^ immature neurons (r = 0.2578, p = 0.0223, Fig. 4F), providing a direct link between this microglial state and a pro-neurogenic environment. Consistent with this, sucrose preference was also positively correlated with Arg1^+^ microglia density (r = 0.6111, p < 0.001, Fig. 4G). Transcriptional profiling confirmed this phenotypic shift, as EA suppressed the CUMS-induced upregulation of pro-inflammatory cytokines (IL6, TNFα) and enhanced the expression of anti-inflammatory and neurotrophic factors (IL4, TGFβ) (Fig. 4H–I). Notably, sham EA had no significant effects on these microglial parameters.

### EA enhances AHN and ameliorates depressive-like behaviors in a microglia-dependent manner

**Fig. 5:**
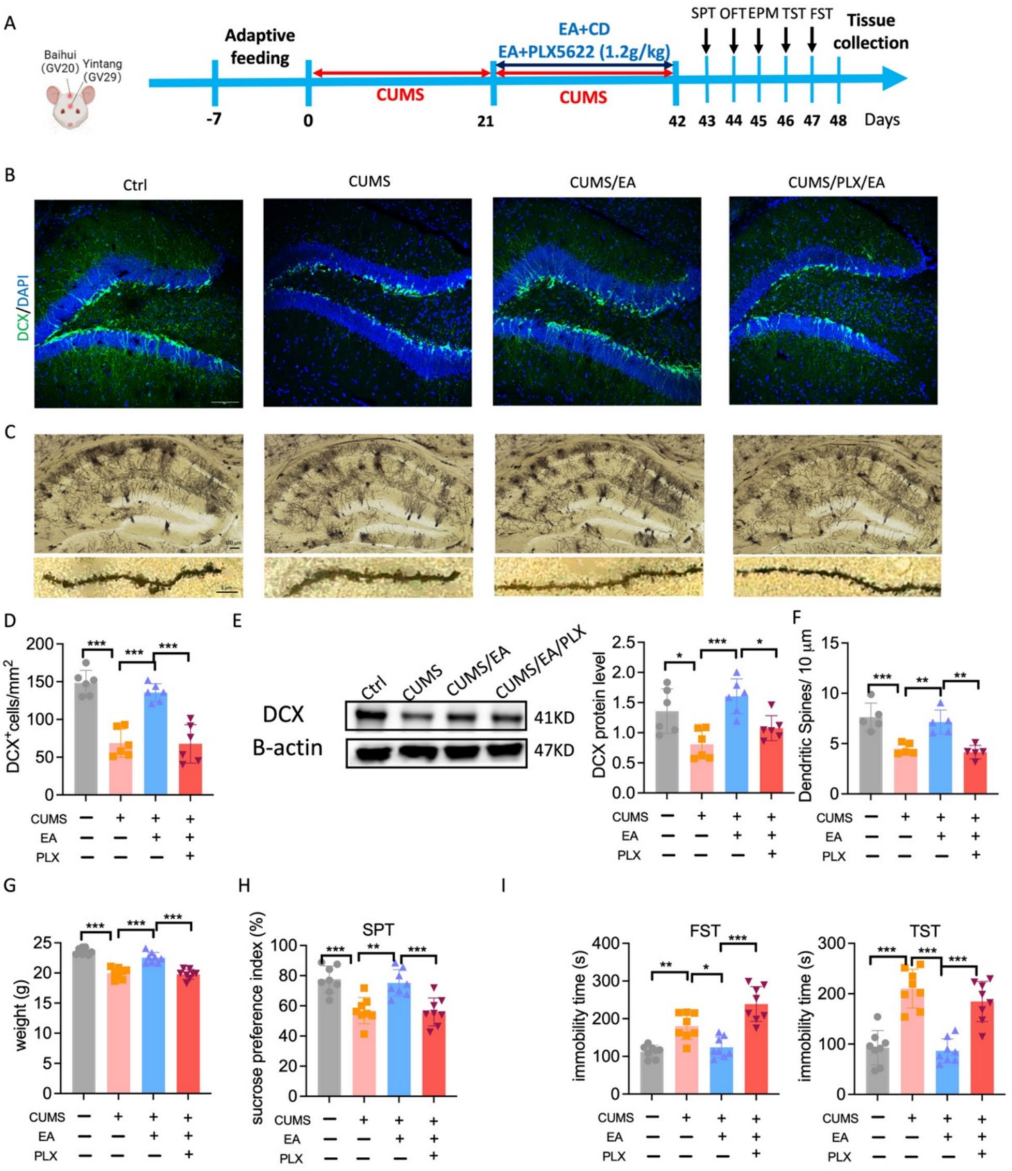
Microglia depletion abrogates the antidepressant and neurogenesis-promoting effects of EA. **A** Timeline of the experimental procedure for assessing adult hippocampal neurogenesis and depressive-like behaviors. **B** Representative confocal images of DCX^+^ cells in DG regions of hippocampus. Scale bars, 100 μm. **C** Representative images of Golgi stain of dendritic spines in the hippocampus and magnified images showing the spine in PLX group. Scale bars, 5 μm. **D** Quantification of the number of DCX^+^ cells in PLX treatment mice, n = 6 mice. **E** Representative immunoblots and quantification of DCX in hippocampus. n = 6 mice. **F** Quantification of spine numbers, n = 25 dendrites from 5 mice/group. **G** Body weight change in CUMS mice after PLX treatment and prior to EA intervention, n = 8 mice. **H** Sucrose preference of CUMS mice treated by EA with or without PLX diet, n = 8 mice. **I** Immobility time of CUMS mice treated by EA with or without PLX diet in FST and TST, n = 8 mice. The values are presented as mean ± SEM. One-way ANOVA with Tukey post hoc test was performed for statistical analysis. *p < 0.05, **p < 0.01, ***p < 0.001. PLX5622, PLX. For detailed F-values, degrees of freedom, and exact p-values of all comparisons, see Supplementary Table S5

To investigate the necessity of microglia for the beneficial effects of EA on neurogenesis and depressive-like behaviors, we depleted microglia using PLX5622 prior to EA treatment (Fig. 5A). Three weeks of PLX5622 treatment effectively eliminated approximately 90% of microglia (Fig. S1A–B). We first assessed hippocampal neurogenesis via DCX immunofluorescence in the DG (Fig. 5B). A one-way ANOVA revealed a significant effect of treatment on the number of DCX⁺ cells (F = 31.18, p < 0.001). Post hoc analysis using Tukey’s test confirmed that CUMS exposure significantly reduced the number of DCX⁺ cells compared to control (p < 0.001). EA treatment effectively reversed this CUMS-induced decrease (p < 0.001). Notably, this pro-neurogenic effect of EA was abolished in mice receiving PLX5622, as the CUMS/EA/PLX group exhibited a significant reduction compared to the CUMS/EA group (p < 0.001) (Fig. 5D). Western blot analysis corroborated these findings at the protein level. A one-way ANOVA indicated a significant effect of treatment on DCX protein expression (F = 8.814, p < 0.001). Post hoc analysis revealed that CUMS significantly downregulated DCX expression compared to control (p < 0.05). EA treatment effectively prevented this CUMS-induced reduction (p < 0.001 vs. CUMS group). Importantly, PLX5622 co-treatment obstructed the effect of EA, as the CUMS/EA/PLX group showed a significant reduction in DCX level compared to the CUMS/EA group (p < 0.05) (Fig. 5E). We subsequently examined the structural and behavioral consequences of microglial ablation. One-way ANOVA across all measures indicated that the beneficial effects of EA were consistently dependent on microglia. Specifically, EA restored the dendritic spine density reduced by CUMS (p < 0.01 vs. CUMS), this effect was abolished by PLX5622 (p < 0.01 vs. CUMS/EA). Behaviorally, the ability of EA to normalize CUMS-induced deficits in weight gain (p < 0.001), sucrose preference (p < 0.01), and immobility time in the FST and TST (p < 0.05, p < 0.001) was prevented in PLX5622-treated mice (p < 0.001 for all comparisons versus CUMS/EA) (Fig. 5F–I).

### EA enhances BDNF signaling through convergent pathways in CUMS-exposed mice

**Fig. 6:**
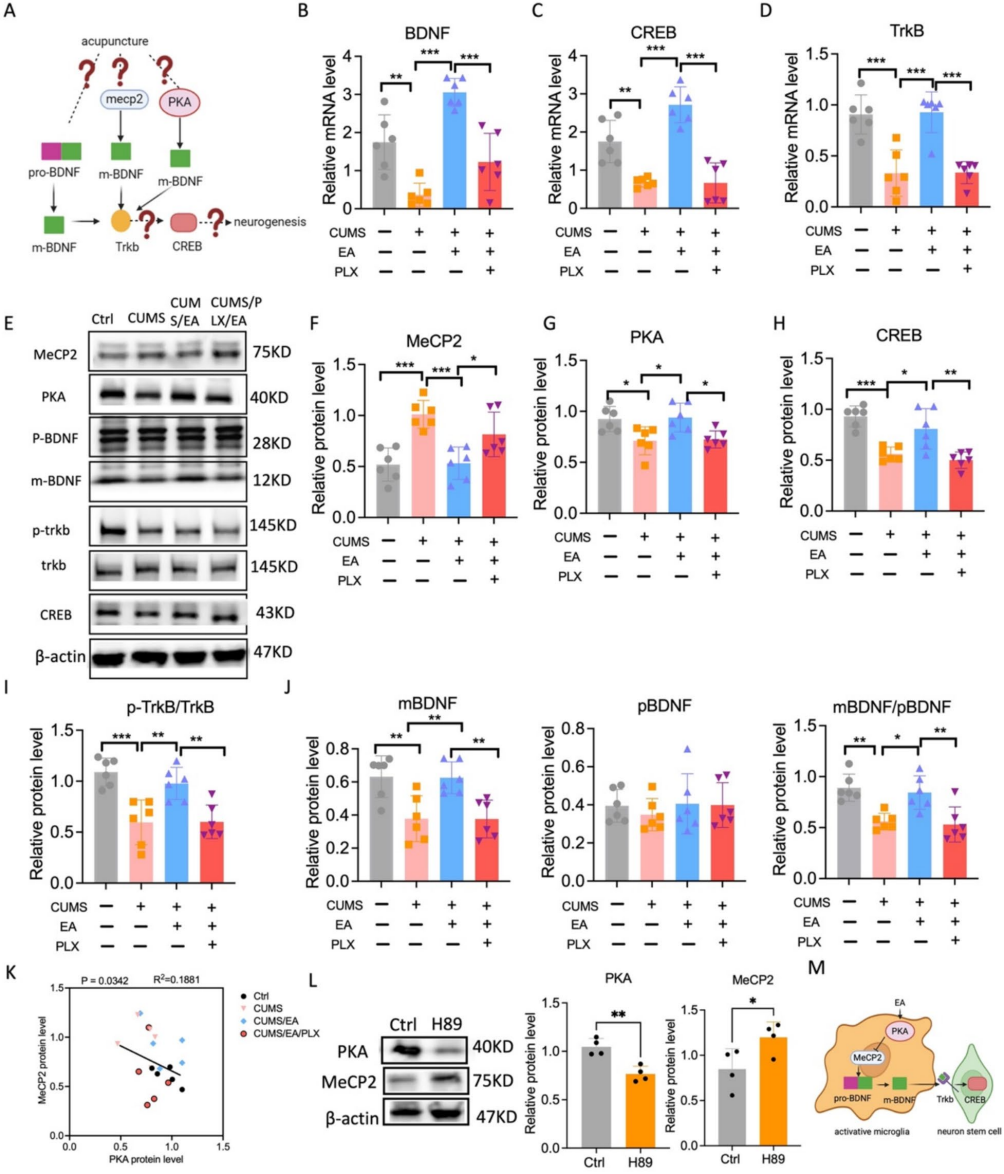
EA increase the BDNF expression in hippocampus of CUMS-exposed mice. **A** The schematic diagram illustrates the upstream and downstream molecules of BDNF. **B**–**D** Quantification of *Bdnf, Creb, Trkb* mRNA level expression, n = 6 mice. **E** Representative immunoblots of BDNF-related molecule in hippocampal extracts. **F**–**G** Quantification of MeCP2, PKA in hippocampus, n = 6 mice. **H** Quantification of CREB, and **I** p-TrkB/TrkB in hippocampus, n = 6 mice. **J** Quantification of pro-BDNF, and mature BDNF, n = 6 mice. The values are presented as mean ± SEM. One-way ANOVA with Tukey post hoc test was performed for statistical analysis. *p < 0.05, **p < 0.01, ***p < 0.001. **K** Correlation between the proportion PKA protein level and MeCP2 protein level in hippocampus, n = 6 mice. **L** Quantification of the protein levels of PKA and MeCP2 in BV2 cells, n = 4 mice. **M** The schematic diagram illustrates the BDNF pathway in microglia and neuron stem cells. For detailed F-values, degrees of freedom, and exact p-values of all comparisons, see Supplementary Table S6

To elucidate the molecular mechanisms through which EA promotes neurogenesis, we systematically examined key pathways both upstream and downstream of BDNF signaling (Fig. 6A). At the transcriptional level, one-way ANOVA indicated that CUMS exposure significantly decreased the mRNA expression of *Bdnf* (F = 28.44, p < 0.001), its receptor *Trkb* (F = 28.14, p < 0.001), and the transcription factor *Creb* (F = 25.60, p < 0.001). Notably, EA reversed the CUMS-induced downregulation of *Bdnf*, *Trkb*, and *Creb* mRNA, effects that were abolished by PLX5622 pretreatment (Fig. 6B–D). Corresponding changes were also observed at the protein level (Fig. 6E). Western blot analysis revealed that EA treatment significantly counteracted the CUMS-induced alterations across multiple regulatory nodes in a microglia-dependent manner. One-way ANOVA revealed a significant effect of treatment on MeCP2 expression (F = 11.54, p < 0.001). Post hoc analysis confirmed that EA downregulated MeCP2 compared to the CUMS group (p < 0.001), an effect that was blocked by PLX5622 pretreatment (p < 0.05; Fig. 6F). Conversely, analysis of PKA expression also indicated a significant treatment effect, with EA increasing PKA levels relative to the CUMS group (p < 0.05). An effect that was likewise prevented by microglia depletion (p < 0.05; Fig. 6G). Furthermore, EA robustly elevated the protein levels of downstream signaling components, with one-way ANOVA showing significant results for both TrkB (F = 13.15, p < 0.001) and phosphorylated CREB (F = 16.21, p < 0.001). Post hoc tests indicated that the enhancements of both TrkB and p-CREB in the EA group were abolished in PLX5622-treated mice (Fig. 6H–I). Finally, we assessed the proteolytic processing of BDNF. Although one-way ANOVA showed that EA did not significantly alter the level of precursor pro-BDNF, it did significantly increase the ratio of mature BDNF (mBDNF) to pro-BDNF (pBDNF) (F = 10.39, p < 0.01). Post hoc analysis confirmed a higher mBDNF/pBDNF ratio in the EA group compared to the CUMS group (p < 0.05), an effect that was abolished by PLX5622 pretreatment (p < 0.01; Fig. 6J). To delineate the relationship between PKA activation and MeCP2 regulation, we first performed a correlation analysis in vivo, which revealed a significant negative relationship between PKA and MeCP2 levels (Fig. 6H). To test whether this correlation reflected a causal relationship, we employed an in vitro pharmacological approach. Treatment of BV2 microglial cells with the PKA inhibitor H89 successfully reduced PKA protein expression and concomitantly increased MeCP2 protein levels (Fig. 6L). This inverse relationship provided direct evidence that PKA acts as an upstream negative regulator of MeCP2. A schematic diagram summarizing this pathway is presented (Fig. 6M). Collectively, these results demonstrate that EA activates a coordinated PKA/MeCP2/BDNF signaling network in microglia to promote hippocampal neurogenesis.

### EA restores the differentiation and mature of NSCs and neuroblasts in mice through BDNF pathway

**Fig. 7:**
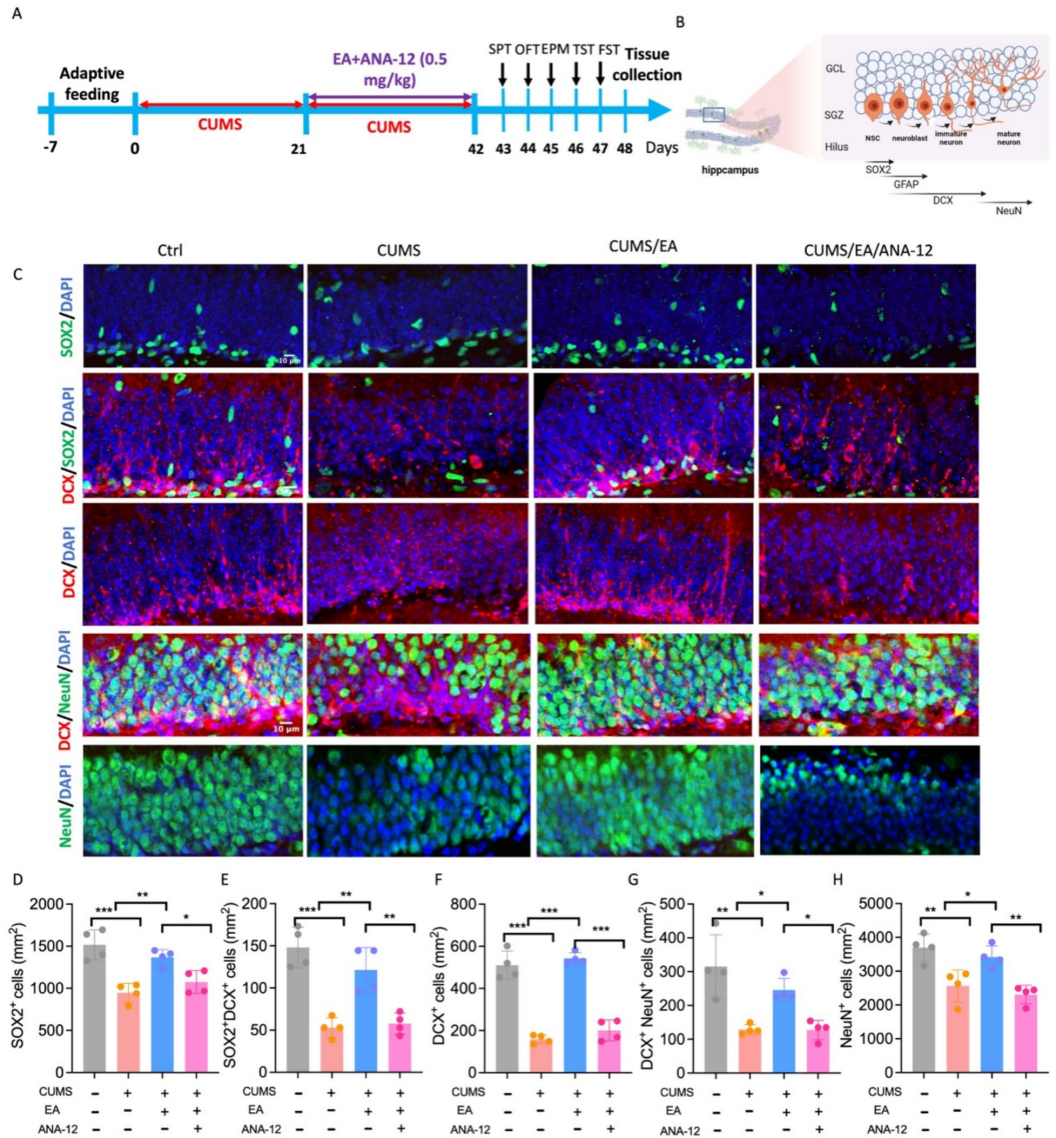
EA enhances AHN in CUMS mice through BDNF pathway. **A** Timeline of the experimental procedure, including ANA-12 administration. **B** Diagram showing the AHN lineage and markers. **C** Representative images of the EA and ANA-12 DG with SOX2 (green), NeuN (green) and DCX (red) cells. Scale bars, 10 μm. **D** Quantification of SOX2^+^ cells, n = 4 mice. **E**. Quantification of SOX2^+^ /DCX^+^ cells, n = 4 mice. **F** Quantification of DCX^+^ cells, n = 4 mice. **G** Quantification of DCX^+^/NeuN^+^ cells, n = 4 mice. **H** Quantification of NeuN^+^ cells, n = 4 mice. The values are presented as mean ± SEM. One-way ANOVA with Tukey post hoc test was performed for statistical analysis. *P < 0.05, **P < 0.01, ***P < 0.001. For detailed F-values, degrees of freedom, and exact p-values of all comparisons, see Supplementary Table S7

To identify the cellular source of BDNF, we conducted multiplex immunofluorescence and analyzed public database. Both methodologies consistently indicated that microglia serve as a primary source of BDNF in the hippocampus (Fig. S2A–D). Quantification of BDNF and the microglial marker IBA1 confirmed that CUMS exposure reduced their co-localization, whereas EA treatment significantly enhanced BDNF-IBA1 co-localization. Conversely, microglial depletion using PLX5622 markedly diminished this signal (Fig. S2E–F). Collectively, these data suggest that microglia play a crucial role in mediating the upregulation of hippocampal BDNF levels following EA treatment. To further investigate whether the BDNF signaling pathway is essential for the protective effects of EA on neurogenesis in CUMS-exposed mice, we inhibited the BDNF receptor TrkB using ANA-12. ANA-12 was administered 30 min prior to EA treatment to block TrkB signaling (Fig. 7A). Given the critical role of AHN in depression, we analyzed various developmental stages of AHN in EA-treated mice. As shown in Fig. 7B and C, NSCs or neural precursor cells (NPCs), neuroblasts, and mature neurons were labeled with SOX2, DCX, and NeuN, respectively. A one-way ANOVA revealed a significant treatment effect on the number of SOX2⁺ cells (F = 19.33, p < 0.001). Post hoc analysis using Tukey’s test confirmed that the number of SOX2⁺ cells was significantly reduced in CUMS mice compared to control (p < 0.001). EA treatment reversed this decrease (p < 0.01 vs. CUMS group), an effect that was abolished by ANA-12 pre-treatment, as the CUMS/EA/ANA-12 group showed a significant reduction compared to the CUMS/EA group (Fig. 7D and Fig. S3A). These results indicate that BDNF/TrkB signaling is necessary for EA-mediated preservation of NSCs. Since NPCs and neuroblasts are derived from NSCs, impairment of NSCs would consequently affect downstream cell populations. A one-way ANOVA revealed a significant treatment effect on the number of SOX2⁺/DCX⁺ cells (F = 22.63, p < 0.001). Post hoc analysis confirmed that CUMS significantly decreased the number of SOX2⁺/DCX⁺ cells compared to control (p < 0.001). EA treatment increased this population (p < 0.01), while ANA-12 pre-treatment blocked the EA-induced increase (p < 0.01; Fig. 7E and Fig. S3B). Similarly, a one-way ANOVA on DCX⁺ cells showed a significant treatment effect (F = 79.36, p < 0.001). The CUMS-induced decrease in DCX⁺ cells (p < 0.001 vs. control) was reversed by EA (p < 0.001 vs. CUMS), and this effect was abolished by ANA-12 (CUMS/EA/ANA-12 vs. CUMS/EA, p < 0.001; Fig. 7F and Fig. S3C). Mature neurons, which are critical for neural circuit function and behavioral outcomes, were also affected. The numbers of DCX⁺/NeuN⁺ cells and NeuN⁺ cells were substantially reduced in CUMS mice. EA treatment restored these populations, and this effect was reversed by ANA-12 (Fig. 7G–H, and Fig. S3D–E). These results suggest that CUMS impairs the differentiation and maturation of NSCs, and that EA mitigates these deficits through a BDNF/TrkB-dependent mechanism.

### ANA-12 reversing EA improves anxiodepressive behavior in CUMS mice

**Fig. 8:**
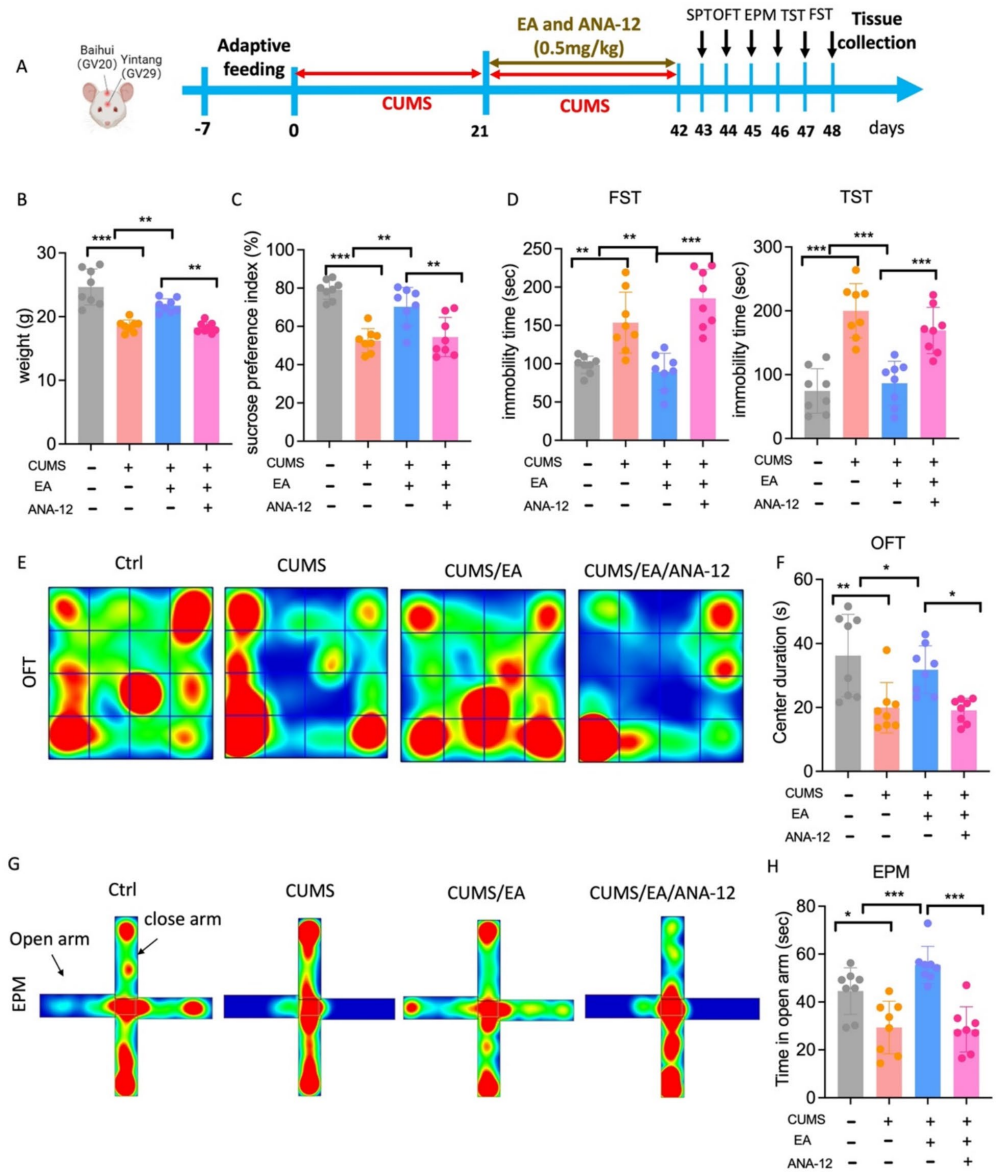
ANA-12 reversing EA improves depression-like behavior in chronic stress mice. **A** Experimental scheme for administration of ANA-12 prior to EA treatment in CUMS-exposed mice. **B** Effect of ANA-12 on the changes of body weight of EA-treated mice, n = 8 mice. **C** Effect of EA on the changes of sucrose preference index of EA-treated mice, n = 8 mice. **D** Mice treated by ANA-12 were assessed the immobility time in FST and TST, n = 8 mice. **E** Heatmap of the trajectory of mice in OFT after ANA-12 administration prior to EA treatment in CUMS-exposed mice. **F** Time spent in center of the open field, n = 8 mice. **G** Representative EPM tracks after ANA-12 administration prior to EA treatment in CUMS-exposed mice. **H** Time spent and in the open arms of the EPM, n = 8 mice. The values are presented as mean ± SEM. One-way ANOVA with Tukey post hoc test was performed for statistical analysis. *P < 0.05, **P < 0.01, ***P < 0.001. For detailed F-values, degrees of freedom, and exact p-values of all comparisons, see Supplementary Table S8

To determine whether BDNF-TrkB signaling is essential for the sustained antidepressant effects of EA at the behavioral level, ANA-12 was administered to inhibit TrkB prior to each EA session throughout the three-week CUMS and treatment period (Fig. 8A). A one‑way ANOVA on body weight showed a significant treatment effect (F = 26.86, p < 0.001). Post hoc analysis using Tukey’s test confirmed that while EA restored the body weight reduction induced by CUMS (p < 0.01 vs. CUMS group), this beneficial effect was negated by ANA-12 co-treatment, as evidenced by a significant reduction in the CUMS/EA/ANA-12 group compared to the CUMS/EA group (p < 0.01; Fig. 8B). Similarly, the improvement in sucrose preference induced by EA was abolished in ANA-12-treated mice (p < 0.01, Fig. 8C). In the FST and TST, the effect of EA in reducing immobility time was significantly reversed by ANA-12 (p < 0.001, Fig. 8D). We further evaluated anxiety-like behaviors using OFT and EPM. Representative movement trajectories in the OFT are depicted (Fig. 8E). EA treatment increased the time spent in the center zone of the OFT, indicating reduced anxiety. However, this effect was blocked by ANA-12 (p < 0.05, Fig. 8F). Consistent with this, EA also increased the time spent in the open arms of the EPM, and again, ANA-12 abolished this anxiolytic effect (p < 0.001, Fig. 8G–H). Collectively, these results demonstrate that ANA-12 effectively reverses the therapeutic benefits of EA on both anxiodepressive behaviors, confirming that the antidepressant mechanism of EA critically relies on BDNF-TrkB signaling.

## Discussion

In this study, we demonstrate that EA at GV20 and GV29 alleviates anxiodepressive behaviors in CUMS-exposed mice. Our finding indicated that EA promotes a pro-neurogenic microglial phenotype in the hippocampus. Furthermore, these reprogrammed microglia enhance BDNF signaling through a defined PKA/MeCP2/BDNF pathway, which subsequently boosts adult hippocampal neurogenesis. The necessity of this pathway was confirmed, as both microglial ablation (using PLX5622) and TrkB blockade (using ANA-12) abolished the pro-neurogenic and antidepressant effects of EA.

Depression and anxiety disorders exhibit a high degree of comorbidity. Population-representative surveys indicate that approximately 51% of individuals diagnosed with MDD concurrently receive a diagnosis of an anxiety disorder within the same year [36]. CUMS model is known to elicit both depressive-like and anxiety-like behaviors in rodents. However, the anxiety component is often overlooked in related research [12, 37]. To evaluate the efficacy of EA against co-occurring depressive and anxiety-like behaviors, we employed a single cohort tested in a fixed sequence with optimized inter-test intervals to minimize stress carryover. Although this design inherently limits the complete dissociation of anxiety and depression measures, our data robustly demonstrate that EA produces significant and concurrent anxiolytic and antidepressant effects in the CUMS model. These findings support the therapeutic potential of EA treatment for this comorbid phenotype.

Clinical neuroimaging studies consistently show reduced hippocampal volume in patients with major depressive disorder. This structural change is widely interpreted as evidence of impaired adult hippocampal neurogenesis, supporting a key pathophysiological link to the disorder [34]. In line with the significance of this mechanism, our prior work demonstrated that active components of traditional Chinese medicine can alleviate depressive-like behaviors by enhancing neurogenesis in the hippocampal dentate gyrus [38]. In the present study, we found that EA increased the populations of neural progenitor cells (DCX⁺/BrdU⁺) and mature neurons (NeuN⁺). This increase in neurogenesis was accompanied by a higher density of dendritic spines, a finding consistent with the improved neuronal integration and functionality previously reported for crocin treatment in the CUMS model [39]. Beyond these neuronal changes, we observed a novel structural phenomenon through which EA may support neurogenesis. It significantly increased direct physical interactions between microglia and DCX⁺ neuroblasts. Notably, the proportion of the most intimate contact morphology (Class I) positively correlated with both the number of immature neurons and the amelioration of anhedonia. These correlations suggest a potential supportive and regulatory role for microglia-neuroblast contact, which may facilitate neuronal maturation and contribute to behavioral recovery. The precise molecular mechanism underlying this interaction warrants future investigation.

Furthermore, we characterized the microglial state under chronic stress and following EA intervention. CUMS exposure induced microglial hyperactivation, characterized by increased cell density, simplified branching morphology, and elevated expression of the phagocytic marker CD68. Remarkably, EA treatment effectively normalized microglial density, restored process complexity, and suppressed CD68 overexpression. These findings indicate a restoration of homeostatic microglial function. Our data further suggest that EA promotes a distinct pro-neurogenic microglial phenotype, characterized by elevated the expression of Arg1. Accumulating evidence indicates that Arg1⁺ microglia play a pivotal role in maintaining CNS homeostasis and facilitating repair [15, 40]. These microglia contribute to an anti-inflammatory and pro-repair microenvironment through dual mechanisms. First, they competitively inhibit inducible nitric oxide synthase, thereby reducing the production of neurotoxic nitric oxide and oxidative stress. Second, they generate polyamines, such as putrescine and spermidine, which are known to support neurogenesis and synaptic plasticity [41, 42]. Thus, the increase in Arg1⁺ microglia following EA represents a functional shift towards a phenotype that not only resolves inflammation but also actively supports the neurogenic niche. This mechanism provides a compelling explanation for how EA-induced microglial remodeling creates a permissive environment conducive to hippocampal neurogenesis.

To definitively establish the causal role of microglia in mediating the antidepressant effects of EA, we employed selective microglial ablation using the CSF1R inhibitor PLX5622. Our results demonstrate that microglial depletion completely abolished the beneficial effects of EA across multiple levels. Specifically, it blocked the EA-induced increase in DCX⁺ neuroblasts, reversed the enhancement in dendritic spine density, and eliminated the improvement in depressive-like behaviors. These findings unequivocally identify microglia as essential regulators of EA-driven functional recovery. This obligatory role positions microglia not merely as bystanders but as central executors in the EA-induced restorative cascade, bridging the treatment’s effects from cellular remodeling to behavioral improvement. While previous studies have reported that EA can restore impaired adult neurogenesis in depression models [37], our work directly establishes a causal link. We demonstrate that depleting this specific cell type abolishes the entire spectrum of EA’s benefits. Consequently, the critical question shifts from whether microglia are involved to how EA recalibrates microglial function to enable this pro-recovery phenotype. This transition in focus is supported by our observed normalization of microglial morphology and activity following EA intervention.

Having established microglia as a key cellular player, we next sought to identify the molecular mediator through which they facilitate neurogenesis. Our investigation pinpointed BDNF signaling as a critical pathway. Although BDNF is classically considered to be primarily neuronal in origin under physiological conditions, emerging evidence underscores the importance of microglia-derived BDNF in mediating synaptic plasticity and neural repair [43, 44]. In the present study, cellular source mapping via multiplex immunofluorescence revealed significant co-localization of BDNF with microglia in the hippocampus. Furthermore, EA treatment markedly enhanced microglial BDNF expression, whereas microglial depletion with PLX5622 substantially reduced BDNF levels, indicating that microglia serve as a major cellular source of BDNF in response to EA. Future studies utilizing microglia-specific BDNF knockout mice will be essential to dissect this specific contribution. While some studies utilizing transgenic models or assessing baseline conditions report predominant neuronal BDNF expression [45]. Our findings under therapeutic intervention align with a growing body of literature highlighting the region- and context-dependent role of glial cells in BDNF production. For instance, previous work has documented BDNF enrichment in the dentate gyrus and CA3 subregions of the hippocampus [46], with glial cells playing a key role in its regulation. Thus, in the context of EA, microglia emerge as functionally indispensable contributors to the BDNF-dependent enhancement of neurogenesis.

Having established that EA restores hippocampal BDNF primarily from microglia, a critical subsequent question is how this upregulation of BDNF translates into functional recovery. Our findings indicate that this translation occurs via the activation of the BDNF/TrkB/CREB signaling cascade, a key pathway known to enhance synaptic plasticity [18, 47]. Beyond this downstream effector pathway, a more comprehensive understanding necessitates examining the upstream events that regulate BDNF availability and activity. Prior studies have often focused on correlative changes in BDNF levels, leaving its multi-layered regulation less explored. In addressing this gap, our study systematically investigates three pivotal upstream mechanisms governing BDNF modulation: (1) the proteolytic cleavage of pro-BDNF into mature BDNF (mBDNF); (2) the transcriptional regulation of BDNF via the epigenetic modulator MeCP2; and (3) PKA-dependent induction of BDNF expression. Utilizing a combination of in vivo and in vitro approaches, we demonstrate that EA robustly facilitates molecular changes across the PKA/MeCP2/BDNF axis, highlighting its multifaceted capacity to regulate BDNF signaling at multiple functional levels. This aligns with established evidence that PKA activation leads to MeCP2 phosphorylation, which in turn relieves transcriptional repression of BDNF, as shown in a fluoxetine-treated mouse model [48]. Our work thus delineates a multi-tiered regulatory model through which EA coordinates BDNF signaling, from its initial transcription and maturation to its downstream functional effects.

The functional contribution of BDNF/TrkB signaling was confirmed by administering the receptor antagonist ANA-12 [49], which effectively blocked the neurogenic and behavioral benefits of EA. Computational docking models indicated that ANA-12 is specifically designed to bind directly and selectively to the TrkB receptor. It potently inhibits BDNF-induced TrkB phosphorylation and downstream signaling without altering the functions of the related receptors TrkA and TrkC, and it binds specifically to the d5 domain of TrkB [50]. Previous studies have demonstrated its ability to cross the blood–brain barrier and inhibit central TrkB activity, without affecting neuronal survival [51]. Although K252a is often referred to as an inhibitor of BDNF/TrkB, it is not a specific inhibitor of TrkB [52]. K252a is a broad-spectrum kinase inhibitor that affects multiple serine/threonine kinases and tyrosine kinases [53]. Therefore, to ensure precise pathway interrogation, ANA-12 was chosen for its high specificity toward TrkB. While absolute specificity is challenging to prove pharmacologically, the convergence of compound selectivity, established dosing, and our functionally coherent results strongly suggest that the critical role of BDNF/TrkB signaling identified here is not an artifact of off-target actions. Our results decisively demonstrate that pharmacological inhibition of TrkB with ANA-12 completely abolished both the pro-neurogenic and antidepressant-like behavioral effects of EA. In summary, our research confirms the involvement of BDNF signaling in the therapeutic effects of EA. Mechanistically, it delineates specific upstream pathways that regulate this response. Furthermore, we provide functional validation through pharmacological inhibition. Together, these findings offer a more comprehensive and mechanistic understanding of how EA acts to combat depression.

## Conclusion

**Fig. 9:**
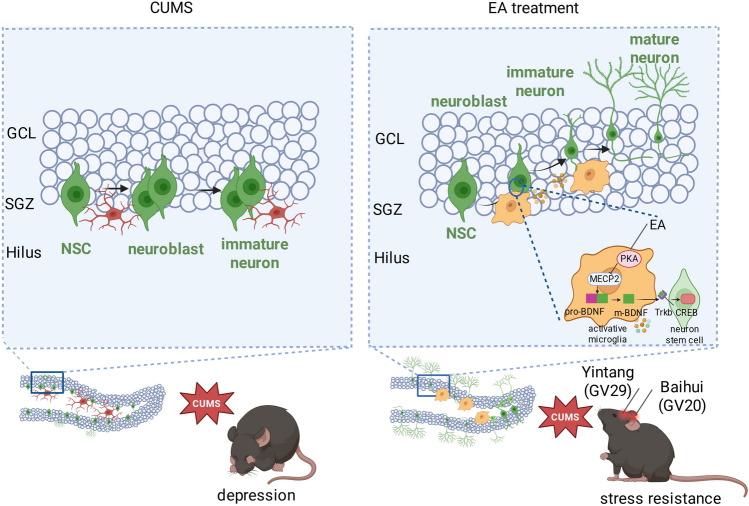
EA alleviates depressive-like behaviors by orchestrating a microglia-centered recovery program. EA reprograms hippocampal microglia towards a pro-neurogenic phenotype. This cellular shift triggers the PKA/MeCP2/BDNF pathway within microglia, elevating bioactive BDNF. The increased BDNF, in turn, activates TrkB signaling on neural precursor cells and mature neurons, thereby enhancing adult hippocampal neurogenesis and synaptic plasticity to mediate functional recovery. The figure was created with BioRender.com

In this study, we demonstrate that EA treatment effectively reverses CUMS-induced anxiodepressive behaviors in mice. Mechanistically, EA induces a pro-neurogenic phenotype in hippocampal microglia and enhances AHN, primarily through activation of the PKA/MeCP2/BDNF pathway (Fig. 9). Our work elucidates a novel immunomodulatory mechanism of EA, highlighting its potential as a multi-target therapeutic strategy for depression.

## Limitations of the study

Although our study delineates a microglia-dependent mechanism through which EA enhances BDNF signaling and promotes neurogenesis, several key mechanistic questions remain. First, a potential limitation lies in our behavioral testing paradigm. While the FST and TST were conducted on separate days, using both tests in the same cohort carries a risk of mutual influence. This is because both tests measure a similar form of “passive coping” behavior. Second, although our data firmly establish that microglia are necessary for the BDNF-mediated effects of EA, we do not definitively distinguish between two plausible cellular mechanisms: (1) that reprogrammed microglia directly upregulate and release BDNF, or (2) that they create a permissive environment (e.g., via anti-inflammatory cytokines) which in turn enables neurons or astrocytes to become the primary source of BDNF. Elucidating this precise cellular crosstalk and the integrated molecular circuitry will be a critical focus of our subsequent research.

## Materials and methods

### Ethics

All animal experiments were approved by the guidelines of Chengdu University of Traditional Chinese Medicine, as well as the revised Animals (Scientific Procedures) Act 1986 (UK) and Directive 2010/63/EU. All animal experiments were also conducted in accordance with the guide of the Laboratory Animal Care and Use Committee of Chengdu University of Traditional Chinese Medicine (procedure code: 2,025,077, 26 May 2025).

### Animals care 

Male C57BL/6 J mice, 8-week-old (supplier: Chengdu Dashuo Biological Technology) were employed to minimize confounding effects of estrogen cycle variability on neurobehavioral outcomes. Mice under controlled environmental conditions (22–24 °C, 45–55% humidity) with ad libitum access to standard chow and filtered water. A standardized 12-h photoperiod (07:00–19:00 illumination) was maintained to prevent circadian rhythm disruptions.

### Study design

We conducted the following series of experiments to comprehensively test our research hypotheses.

### Experiment 1

To investigate whether EA improves depressive-like behaviors in CUMS mice, mice were randomly assigned to five groups: Control (n = 8); CUMS (n = 10); CUMS/EA (n = 9); CUMS/sham (n = 8); CUMS/IMI (n = 9). Pharmacological intervention involved daily intraperitoneal injections of imipramine (IMI, Sigma-Aldrich, # 113-52-0, 10 mg/kg), following established protocols with dose optimization [40].

### Experiment 2

To determine whether the antidepressant effects of EA depend on microglia, mice were divided into four groups: Control (n = 8); CUMS (n = 8); CUMS/EA (n = 8); CUMS/EA/PLX (n = 8). To pharmacological ablate brain microglia, mice were fed PLX5622-formulated AIN-76A diet (1.2 g PLX5622 per kilogram of diet, Plexxikon) ad libitum. Control mice received a standard AIN-76A diet.

### Experiment 3

To explore the role of BDNF signaling in the therapeutic effects of EA, mice were allocated to four groups: Control (n = 8); CUMS (n = 8); CUMS/EA (n = 8); CUMS/EA/ANA-12 (n = 8). Mice in the CUMS/EA/ANA-12 group were administered ANA-12 (MCE, 0.5 mg/kg) [54] via intraperitoneal injection 30 min prior to each EA session. The ANA-12 was dissolved in a PBS solution containing 10% DMSO.

### Chronic unpredictable mild stress (CUMS) procedure

The CUMS protocol was optimized from an established rodent depression model [38] to induce behavioral and neurobiological deficits. Stressors were administered in a randomized daily sequence and included: 24-h continuous light exposure; 12-h water/food restriction; 45° cage inclination for 12 h; 4-h confinement in ventilated tubes; 5-min exposure to 45 ℃ ambient heat or forced swimming in 0℃ water. CUMS protocol was administered throughout the entire 6-week period. During the 3-week induction phase, mice received 1–2 randomized stressors per day. In the subsequent 3-week intervention phase, a single daily stressor was maintained, concurrent with EA or drug treatment. Behavioral assessments were conducted at two time points: at the end of the initial 3-week stress period (baseline) and again at the end of the 6-week period (post-treatment). At each time point, behavioral tests were distributed over several days while the CUMS (and during the intervention phase, EA) protocol continued. Crucially, on any given testing day, the behavioral assay was scheduled at least 24 h after the last stressor to avoid measuring acute stress responses.

### EA intervention

Following three-week of CUMS induction, mice received standardized EA therapy during the light cycle (09:00–11:00) over a three-week regimen (5 sessions/week). EA was applied at Yintang (GV29; located in the midline depression between the nasal apex and the superior labial margin) and Baihui (GV20; located at the vertex of the head, at the intersection of the sagittal midline and the line connecting the auricular apices), respectively. The acupuncture needles (Suzhou Shenlong Medical, ∅ 0.25 mm × 13 mm) were inserted 2–3 mm deep into the acupoint and connected to the EA instrument (Hwato, SDZ‐V, Suzhou Medical Instruments Co Ltd., Suzhou, China). Then, Han’s acupoint nerve stimulator was used to deliver a dense-disperse wave at frequency of 2/15 Hz (2 Hz for 5 s and 15 Hz for 9 s shifting automatically) and an intensity of 1 mA for 3 weeks (20 min/day, 6 days/week) [55]. For sham EA treatment, acupuncture needles were inserted 0.5 mm lateral to GV20 and GV29 without electrical stimulation. Sham EA design was chosen to specifically control for the potential non-specific effects of needle insertion itself, such as minor tissue injury or a placebo-like effect.

### Behavioral testing timeline and order

Prior to behavioral testing, all mice were handled for three consecutive days to reduce handling stress. On each test day, mice were transferred to the testing room 1 h in advance for acclimation. To minimize stress carryover effects, tests were administered in a sequence progressing from the least to the most aversive paradigm: sucrose preference test (SPT) → open field test (OFT) → elevated plus maze (EPM) → tail suspension test (TST) → forced swim test (FST). A minimum interval of 24 h was maintained between any two tests, a duration established in the literature as sufficient for stress hormone levels to return to baseline [56].

### Sucrose preference test (SPT)

Sucrose preference was assessed using a two-bottle free-choice paradigm according to the CUMS protocol. After 24 h of water and food deprivation, mice were provided with two separate bottles of the same size: one containing a 1% sucrose solution and the other containing tap water. The containers were weighed and the fluid consumption after 2 h was calculated. Sucrose preference = Sucrose consumption/(Sucrose consumption + water consumption) × 100.

### Tail suspension test (TST)

The TST is a desperation behaviors test that assesses the immobility time of mice under uninvited conditions. The tail of mouse was suspended at a height of 20–25 cm by wrapping a piece of tape around the tail, 1 cm from the tip of tail. Each mouse was suspended with the end of its tail for 6 min in a dark box with little background. Duration of immobility, recorded by a video camera (VisuTrack, shanghai, XR-VT), was monitored during the last 4 min of suspension. In addition, mouse was ensured to be undisturbed throughout the test.

### Force swimming test (FST)

The FST assesses the degree of desperation behaviors in an inescapable space. Each mouse was filmed in a transparent acrylic cylinder (High: 25 cm, Depth: 10 cm) filled with 800 mL water (23–25 °C). All trials were filmed by video camera (VisuTrack, shanghai, XR-VT). Immobility was defined as the animal making minimal movement to keep its head out of the water, and a competent blind observer scored immobility. The total immobility time during the last 4 min of test was the main indicator analyzed.

### Open field test (OFT)

The OFT assesses general locomotor activity and anxiety-like behavior. Locomotor activity was recorded for 5 min in a white, rectangular open-field arena (50 × 50 × 50 cm) using video tracking software (VisuTrack, Shanghai, XR-VT). The total distance traveled, number of zone transitions, and time spent in the central zone were analyzed.

### Elevated plus maze (EPM)

The EPM is a widely used to assess the anxiety behaviors. The apparatus consisted of two closed arms (30 cm × 5 cm × 15 cm) and two open arms (30 cm × 5 cm). Each mouse was placed in the apparatus for 5 min. Locomotion was recorded for 5 min using video tracking software (VisuTrack, shanghai, XR-VT). The time spent in open arms, the distance the mice moved, and the number of times into the open arms were analyzed.

### Real-time quantitative fluorescence PCR (qRT-PCR)

Total RNA was extracted from brain lysate using FastPure tissue total RNA isolation kit (Vazyme, #RC112-01) following the protocol. First-strand cDNA synthesis was performed with 1 μg total RNA in a 20 μL reaction volume using HiScript IV All-in-One Ultra RT SuperMix for qPCR Reagent (vazyme, Cat# R433-01). For quantitative gene expression profiling, real-time PCR amplifications were conducted in technical triplicates using FastStart™ SYBR Green Master Mix (Vazyme, Q511-02). Relative transcript levels were calculated via the comparative ΔΔCt method with normalization to β-actin as the endogenous reference. Primer sequences are detailed in Supplementary Table S9.

### Immunofluorescence

The hippocampus was separated and immersed in 4% paraformaldehyde for 12 h before being embedded in paraffin and sectioned, and immersed in 30% sucrose solution until the tissue was sunken to the bottom of the tube. Each sample was cut into a 35 μm thick section (RWD, #FS800). The sections were incubated in 0.01 M phosphate-buffer saline (PBS), washed with PBS Tween-20 (PBST) for three times and blocked with 5% normal donkey serum in 0.05% PBST for 2 h at room temperature, then the sections were incubated in primary antibodies overnight. The secondary antibody incubated for 2 h at room temperature. The primary antibody and second antibody are detailed in Additional file Table S10.

### Western blotting

Cerebral tissue samples were mechanically lysed in ice-cold RIPA buffer (Thermo Fisher, #89,900) containing protease/phosphatase inhibitors (Thermo Fisher, #78,440). Protein quantification was performed using a bicinchoninic acid (BCA) assay kit (Thermo Fisher, #23,225), with equalized lysates resolved on 4–12% gradient Bis–Tris polyacrylamide gels (Thermo Fisher, #NP0322BOX). Electrophoretically separated proteins were transferred onto PVDF membranes (0.45 μm pore size; Millipore, #IPVH00010) under semi-dry conditions. Membranes were blocked with 5% non-fat milk and sequentially incubated with primary antibodies overnight at 4 °C in TBST with gentle agitation, and then HRP-conjugated 2-h incubation at RT. Chemiluminescent signals were developed using ECL substrate (Millipore, #WBKLS0500) and quantified densitometrically via ImageJ (NIH). The primary antibody and second antibody are detailed in Supplementary Table S10. Full, uncropped western blot images showed in figure S4.

### Cell culture and drug treatment

BV2 microglial cells, kindly provided by Professor Zili You from the University of Electronic Science and Technology of China, and cultured in DMEM medium (Gibco, USA) containing 10% fetal bovine serum (FBS, Gibco) under 37 °C and 5% CO_2_. BV2 cells were treated with the PKA inhibitor H89 (5 μM; MedChemExpress, HY-15979) for 5 h. The dose for H89 was based on previous studies [57].

### Golgi staining

Golgi staining was performed using the FD Rapid Golgi Stain™ kit (FD Neuro Technologies, PK401) according to the manufacturer’s instructions. In brief, freshly dissected mouse brains were immersed in an impregnation solution (a mixture of equal volumes of Solutions A and B) and stored in the dark at room temperature for 2 weeks. The brains were then transferred into Solution C and stored under the same conditions for an additional 72 h. Subsequently, the impregnated tissues were sectioned coronally at a thickness of 100 µm using a freezing microtome (Leica CM1950, Germany). The sections were mounted onto gelatin-coated slides with Solution C and subsequently stained by applying Solutions D and E according to the kit protocol. For each mouse, 13–20 dendritic segments were randomly selected for spine density quantification. Dendritic spines were counted and analyzed using ImageJ software.

### Sholl analysis

Z-stack confocal images were processed using the Fiji/ImageJ platform (NIH). Maximum intensity projections were generated from raw stacks, followed by manual selection of microglia with processes fully contained within the imaging field (8–12 cells per field). Individual microglial arbors were isolated via region-of-interest (ROI) cropping and subjected to adaptive thresholding (Otsu method) to create binary masks of process arbors. Artifacts were removed through median filtering (3 × 3 kernel), and morphological complexity was quantified using the Sholl Analysis Plugin (v3.0) with concentric radii spaced at 5 μm intervals.

### Data analysis and statistics

All data analyses were performed in a blinded manner. Statistical analyses were conducted using GraphPad Prism. Data are presented as the mean ± SEM, and the number of independent replicates (n) is specified in the figure legends. The normality of data distribution was assessed using the D’Agostino-Pearson test. For longitudinal behavioral data (e.g., body weight and sucrose preference), a two-way repeated-measures ANOVA was applied, with the Greenhouse–Geisser correction used when the assumption of sphericity was violated (as indicated by Mauchly’s test). Microglial morphology data from Sholl analysis were analyzed using two-way repeated-measures ANOVA, followed by Tukey’s post hoc test. For comparisons among multiple groups at a single time point, one-way ANOVA with Tukey’s post hoc test was used for normally distributed data. The relationship between two continuous variables was assessed using Pearson’s correlation. Statistical significance was defined as p < 0.05, p < 0.01, p < 0.001.

## Supplementary Information

Supplementary Material 1. Supplement Fig. 1. Detection of the efficiency of PLX5622 in eliminating microglia of hippocampus. **A** Representative confocal overview images of microglia in hippocampus after pretreatment with PLX5622 on day 21. Scale bar 100 µm. **B** Quantitative analysis of Iba1-positive microglia in hippocampus at day 21 following AIN or PLX5622 treatment, n = 4. Data are presented as mean ± SEM. two-tailed unpaired Student’s t-test. *p < 0.05, **p < 0.01, ***p < 0.001

Supplementary Material 2. Supplement Fig. 2. Cellular sources and regional expression of BDNF in the mouse brain. **A** Representative fluorescence images showing the co-localization of BDNF (red) with microglial (IBA1, green), astrocytic (GFAP, green), and neuronal (NeuN, green) markers in the hippocampal formation. Scale bar, 20 μm, 5 μm. **B** Representative images from a public database showing the expression pattern of *Bdnf* mRNA in the mouse hippocampal formation, as detected by in situ hybridization (ISH) and Nissl staining (NISS). Scale bar, 100 μm. Data were derived from the Allen Mouse Brain Atlas (mouse.brain-map.org). **C** Single-cell RNA sequencing data from a public database demonstrating the expression of BDNF in microglia isolated from the mouse hippocampus. Data were obtained from the ABC atlas (https://abc.sklehabc.com/). **D** A public database demonstrating the expression of BDNF in other brain. (https://abc.sklehabc.com/). **E** Representative fluorescence images illustrating BDNF (red) and microglial (IBA1, green) co-localization in the hippocampus across different experimental groups. Scale bar, 100 μm. **F** Quantitative analysis of the co-localization of BDNF with IBA1 in the hippocampus across groups. Data are presented as mean ± SEM (n = 6 mice per group). *P < 0.05, **P < 0.01, ***P < 0.001 (one-way ANOVA with Tukey’s post hoc test)

Supplementary Material 3. Supplement Fig. 3. ANA-12 block AHN in hippocampus of EA-treated mice. **A** Representative images of SOX2^+^ cell in DG area. **B** Representative images of SOX2^+^/DCX^+^ cell in DG area. **C** Representative images of DCX^+^ cell in DG area. **E** Representative images of DCX^+^/NeuN^+^ cell in DG area. **F** Representative images of NeuN^+^ cell in DG area. Scale bars, 100 μm

Supplementary Material 4. Fig. S4. Full, uncropped western blot images

Supplementary Material 5. Table S1-8. Detailed statistical analysis for all data presented in Fig. 1–8

Supplementary Material 6. Table S9. PCR primers

Supplementary Material 7. Table S10. List of primary and secondary antibodies

## Data Availability

The data supporting the findings of this study are available within the article and the supplementary materials.

## Abbreviations

EA: Electroacupuncture
GV29: Yintang
GV20: Baihui
CUMS: Chronic unpredictable mild stress
SPT: Sucrose preference test
FST: Forced swimming test
TST: Tail suspension test
EPM: Elevated plus maze
OFT: Open field test
BDNF: Brain-derived neurotrophic factor
CSF-1R: Colony-stimulating factor-1 receptor
MDD: Major depressive disorder
AHN: Adult hippocampal neurogenesis
NSC: Neural stem cells
DG: Dentate gyrus
TrkB: Tropomyosin receptor kinase B
CREB: CAMP response element-binding
MeCP2: Methyl-CpG binding protein 2
NPC: Neural precursor cells
