# Supplementary figures and images for: Electroacupuncture promotes BDNF-dependent neurogenesis via microglial reprogramming in a chronic stress model
Source: Chin Med. 2026 Feb 3;21:62. doi: 10.1186/s13020-026-01334-y (PMC12866076; doi:10.1186/s13020-026-01334-y)

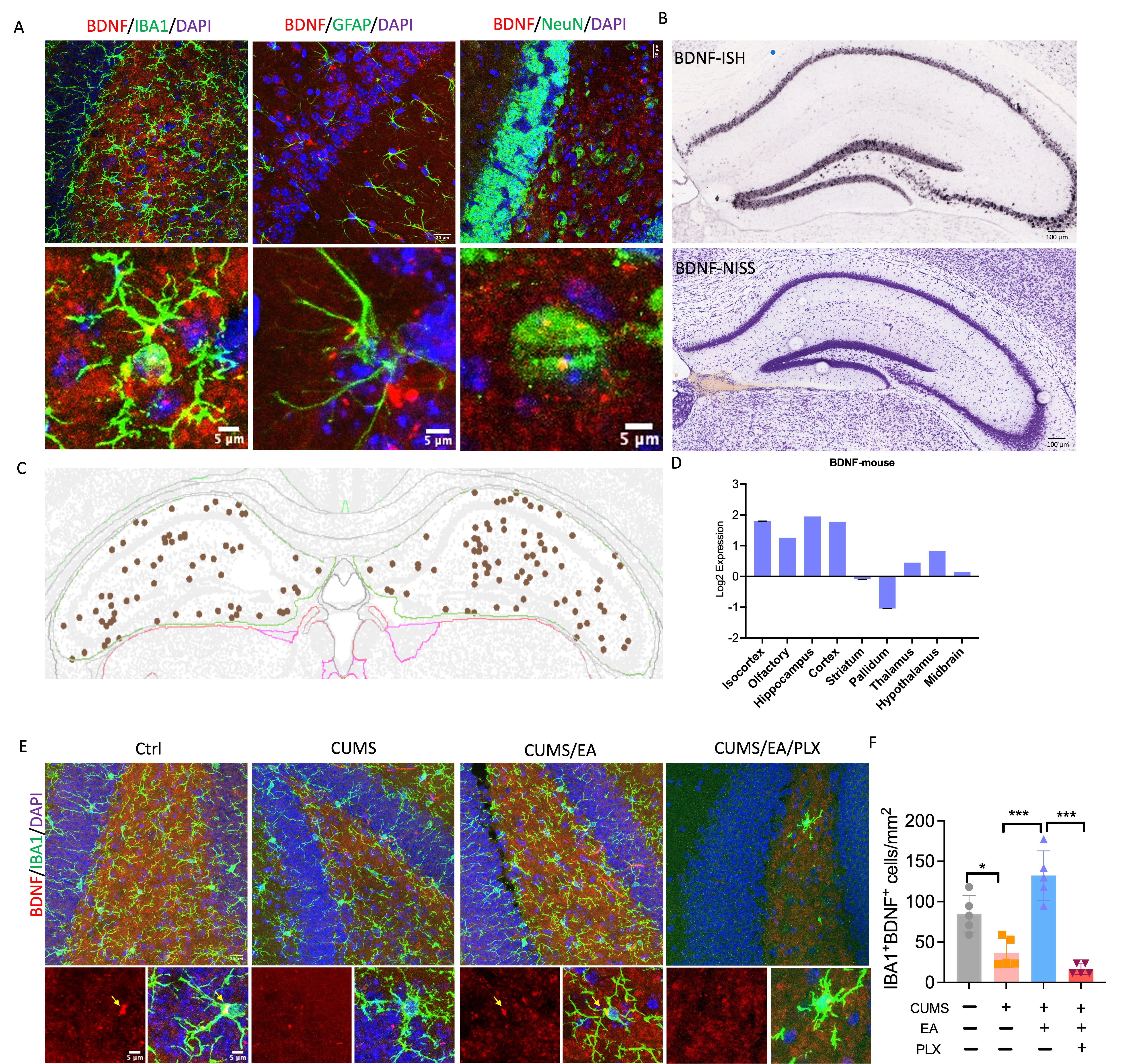

Supplement: Supplementary file 1 — Supplementary Material 1. Supplement Fig. 1. Detection of the efficiency of PLX5622 in eliminating microglia of hippocampus. A Representative confocal overview images of microglia in hippocampus after pretreatment with PLX5622 on day 21. Scale bar 100 µm. B Quantitative analysis of Iba1-positive microglia in hippocampus at day 21 following AIN or PLX5622 treatment, n = 4. Data are presented as mean ± SEM. two-tailed unpaired Student’s t-test. *p < 0.05, **p < 0.01, ***p < 0.001 [file 13020_2026_1334_MOESM1_ESM.jpg]

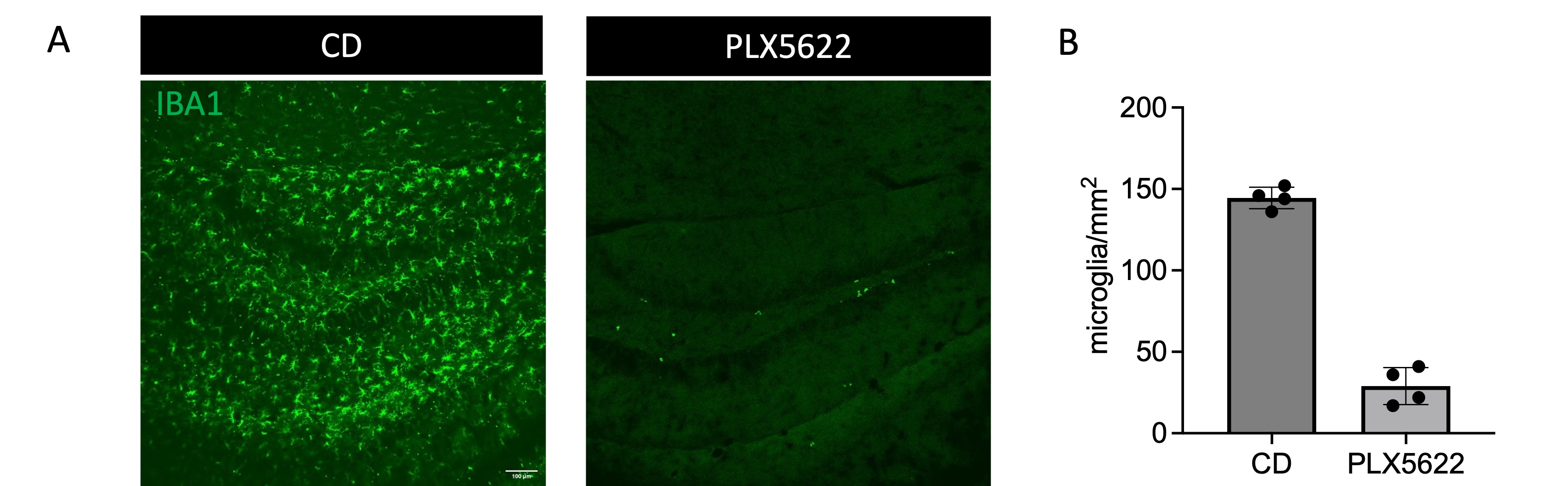

Supplement: Supplementary file 2 — Supplementary Material 2. Supplement Fig. 2. Cellular sources and regional expression of BDNF in the mouse brain. A Representative fluorescence images showing the co-localization of BDNF (red) with microglial (IBA1, green), astrocytic (GFAP, green), and neuronal (NeuN, green) markers in the hippocampal formation. Scale bar, 20 μm, 5 μm. B Representative images from a public database showing the expression pattern of Bdnf mRNA in the mouse hippocampal formation, as detected by in situ hybridization (ISH) and Nissl staining (NISS). Scale bar, 100 μm. Data were derived from the Allen Mouse Brain Atlas (mouse.brain-map.org). C Single-cell RNA sequencing data from a public database demonstrating the expression of BDNF in microglia isolated from the mouse hippocampus. Data were obtained from the ABC atlas (https://abc.sklehabc.com/). D A public database demonstrating the expression of BDNF in other brain. (https://abc.sklehabc.com/). E Representative fluorescence images illustrating BDNF (red) and microglial (IBA1, green) co-localization in the hippocampus across different experimental groups. Scale bar, 100 μm. F Quantitative analysis of the co-localization of BDNF with IBA1 in the hippocampus across groups. Data are presented as mean ± SEM (n = 6 mice per group). *P < 0.05, **P < 0.01, ***P < 0.001 (one-way ANOVA with Tukey’s post hoc test) [file 13020_2026_1334_MOESM2_ESM.jpg]

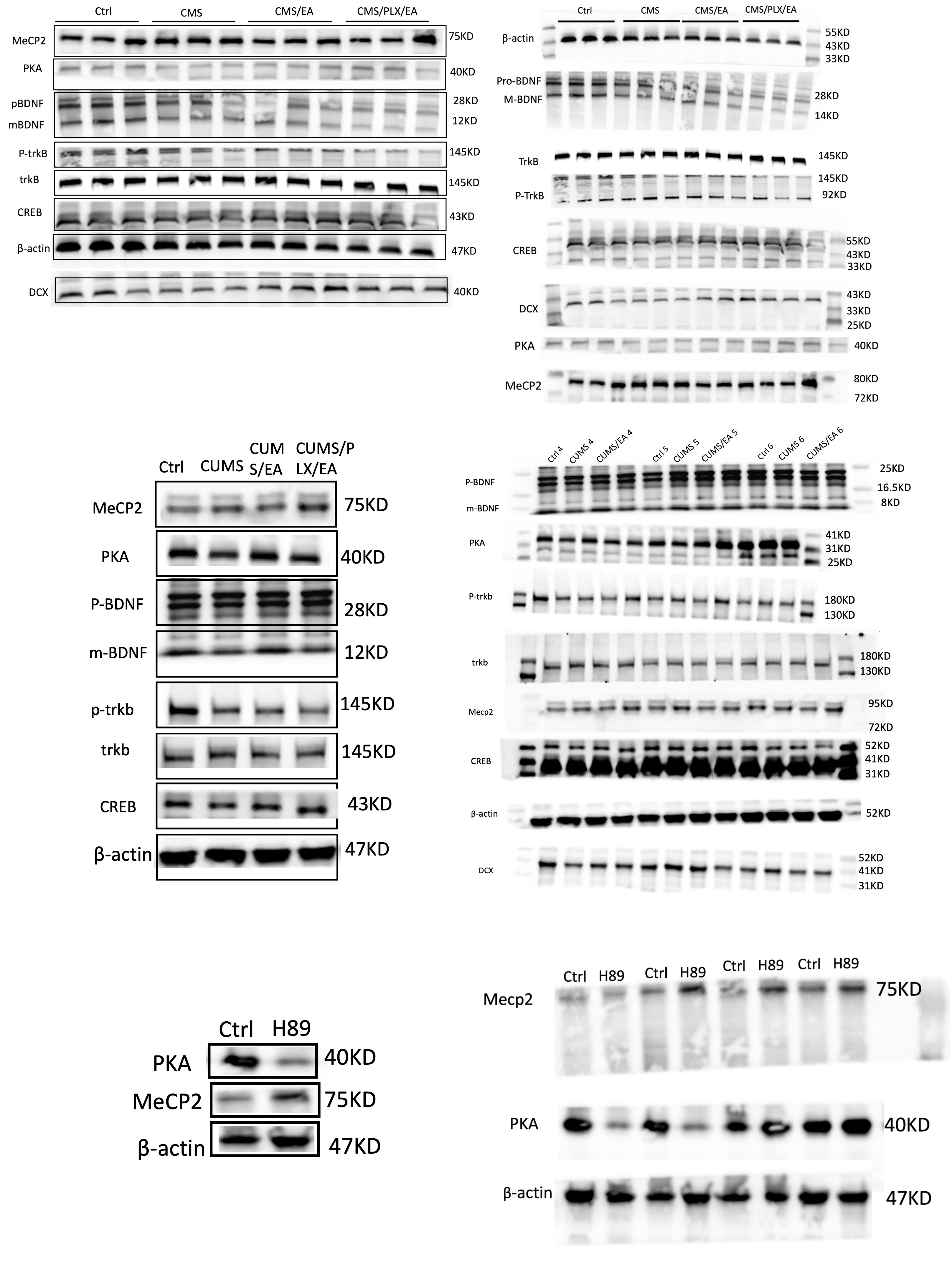

Supplement: Supplementary file 3 — Supplementary Material 3. Supplement Fig. 3. ANA-12 block AHN in hippocampus of EA-treated mice. A Representative images of SOX2+ cell in DG area. B Representative images of SOX2+/DCX+ cell in DG area. C Representative images of DCX+ cell in DG area. E Representative images of DCX+/NeuN+ cell in DG area. F Representative images of NeuN+ cell in DG area. Scale bars, 100 μm [file 13020_2026_1334_MOESM3_ESM.jpg]

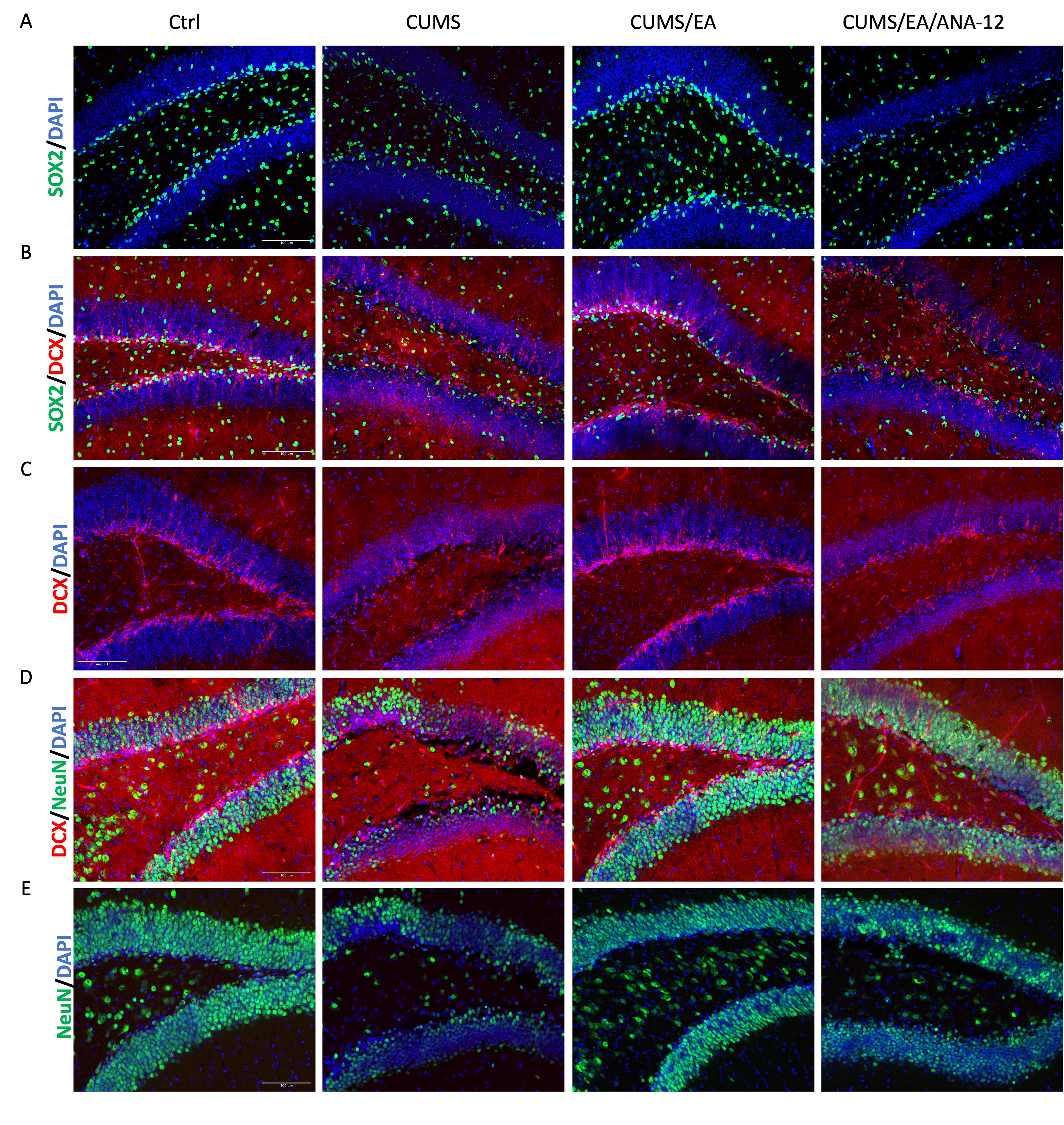

Supplement: Supplementary file 4 — Supplementary Material 4. Fig. S4. Full, uncropped western blot images [file 13020_2026_1334_MOESM4_ESM.jpg]
