# Supplementary material for: Electroacupuncture promotes BDNF-dependent neurogenesis via microglial reprogramming in a chronic stress model
Source: Chin Med. 2026 Feb 3;21:62. doi: 10.1186/s13020-026-01334-y (PMC12866076; doi:10.1186/s13020-026-01334-y)
Supplement: Supplementary file 5 — Supplementary Material 5. Table S1-8. Detailed statistical analysis for all data presented in Fig. 1–8 [file 13020_2026_1334_MOESM5_ESM.docx]

Table S1. The statistical methods and data in Figure 1

| **Figure** | **Statistical Test** | | | **Main Effect / Interaction** | **F value (df)** | **p value** | **Post-hoc Comparison** | **Post-hoc p value** |  |
| --- | --- | --- | --- | --- | --- | --- | --- | --- | --- |
| Fig. 1B | Two-way ANOVA | | | Time **×**Treatment | F (24, 264) = 5.335 | p<0.0001 | Control vs. CUMS | p=0.0001 |  |
|  |  | | | Time | F (3.479, 153.1) = 21.84 | p<0.0001 | CUMS vs. CUMS+EA | p=0.0010 |  |
|  |  | | | Treatment | F (4, 44) = 14.34 | p<0.0001 | CUMS vs. CUMS+IMI | p=0.0023 |  |
| Fig. 1C | Two-way ANOVA | | | Time **×**Treatment | F (24, 252) = 11.33 | p<0.0001 | Control vs. CUMS | p<0.0001 |  |
|  |  | | | Time | F (5.078, 213.3) = 103.8 | p<0.0001 | CUMS vs. CUMS+EA | p=0.0005 |  |
|  | | | |  | Treatment | F (4, 42) = 28.25 | p<0.0001 | CUMS vs. CUMS+IMI | p<0.0001 |
| Fig. 1D | | | Two-way ANOVA | | Time **×**Treatment | F (8, 80) = 18.21 | p<0.0001 | Control vs. CUMS | p<0.0001 |
|  | | | |  | Time | F (1.864, 74.57) = 88.91 | p<0.0001 | CUMS vs. CUMS+EA | p<0.0001 |
|  | | | |  | Treatment | F (4, 40) = 21.01 | p<0.0001 | CUMS vs. CUMS+IMI | p<0.0001 |
| Fig. 1E | | | | Two-way ANOVA | Time **×**Treatment | F (8, 66) = 7.031 | p<0.0001 | Control vs. CUMS | p<0.0001 |
|  | | | |  | Time | F (2, 66) = 66.57 | p<0.0001 | CUMS vs. CUMS+EA | p=0.0026 |
|  | | | |  | Treatment | F (4, 66) = 8.491 | p<0.0001 | CUMS vs. CUMS+IMI | p=0.0468 |
|  | | | |  |  |  |  |  |  |
|  | | | |  |  |  |  |  |  |

Note: The 6-week time point was chosen for detailed mechanistic investigations because the behavioral deficits and the proposed molecular pathways were most prominently manifested and modulated at this endpoint.

Table S2. The statistical methods and data in Figure 2

| **Figure** | **Statistical Test** | | | **Main Effect / Interaction** | **F value (df)** | **p value** | **Post-hoc Comparison** | **Post-hoc p value** |  |
| --- | --- | --- | --- | --- | --- | --- | --- | --- | --- |
| Fig.2B | One-way ANOVA | | | Group Effect | F (4, 39) = 0.8017 | p=0.5315 | Control vs. CUMS | p=0.4923 |  |
|  |  | | |  |  |  | CUMS vs. CUMS+EA | p>0.9999 |  |
|  |  | | |  |  |  | CUMS vs. CUMS+IMI | p=0.9865 |  |
| Fig.2C | One-way ANOVA | | | Group Effect | F (4, 39) = 9.850 | p<0.0001 | Control vs. CUMS | p=0.0101 |  |
|  |  | | |  |  |  | CUMS vs. CUMS+EA | p=0.0062 |  |
|  |  | | |  |  |  | CUMS vs. CUMS+IMI | p=0.0108 |  |
| Fig.2D | One-way ANOVA | | | Group Effect | F (4, 39) = 21.44 | p<0.0001 | Control vs. CUMS | p<0.0001 |  |
|  |  | | |  |  |  | CUMS vs. CUMS+EA | p<0.0001 |  |
|  | | | |  |  |  |  | CUMS vs. CUMS+IMI | p=0.0212 |
| Fig.2E | | | One-way ANOVA | | Group Effect | F (4, 39) = 15.42 | p<0.0001 | Control vs. CUMS | p<0.0001 |
|  | | | |  |  |  |  | CUMS vs. CUMS+EA | p=0.0012 |
|  | | | |  |  |  |  | CUMS vs. CUMS+IMI | p=0.0126 |
| Fig. 2G | | | | One-way ANOVA | Group Effect | F (4, 39) = 0.5506 | p=0.5506 | Control vs. CUMS | p=0.7000 |
|  | | | |  |  |  |  | CUMS vs. CUMS+EA | p=0.6315 |
|  | | | |  |  |  |  | CUMS vs. CUMS+IMI | p=0.9994 |
| Fig. 2H | | | | One-way ANOVA | Group Effect | F (4, 39) = 0.4412 | p=0.7781 | Control vs. CUMS | p=0.7451 |
|  | | | |  |  |  |  | CUMS vs. CUMS+EA | p>0.9999 |
|  | | | |  |  |  |  | CUMS vs. CUMS+IMI | p=0.9986 |
| Fig. 2I | | | | One-way ANOVA | Group Effect | F (4, 39) = 14.21 | p<0.0001 | Control vs. CUMS | p<0.0001 |
|  | | | |  |  |  |  | CUMS vs. CUMS+EA | p=0.0024 |
|  | | | |  |  |  |  | CUMS vs. CUMS+IMI | p=0.0175 |
| Fig. 2J | | | | One-way ANOVA | Group Effect | F (4, 39) = 10.79 | p<0.0001 | Control vs. CUMS | p=0.0037 |
|  | | | |  |  |  |  | CUMS vs. CUMS+EA | p=0.0022 |
|  | | | |  |  |  |  | CUMS vs. CUMS+IMI | p=0.0398 |

Table S3. The statistical methods and data in Figure 3

| **Figure** | **Statistical Test** | | **Main Effect / Interaction** | **F value (df)** | **p value** | **Post-hoc Comparison** | **Post-hoc p value** |  |
| --- | --- | --- | --- | --- | --- | --- | --- | --- |
| Fig. 3C | One-way ANOVA | | Group Effect | F (3, 16) = 12.99 | p<0.0001 | Control vs. CUMS | p=0.0002 |  |
|  |  | |  |  |  | CUMS vs. CUMS+EA | p=0.0009 |  |
|  | | |  |  |  |  | CUMS vs. CUMS+sham | p=0.4391 |
| Fig.  3D | | | One-way ANOVA | Group Effect | F (3, 16) = 9.777 | p=0.0007 | Control vs. CUMS | p=0.0033 |
|  | | |  |  |  |  | CUMS vs. CUMS+EA | p=0.0328 |
|  | | |  |  |  |  | CUMS vs. CUMS+sham | p=0.8245 |
| Fig.  3E | | | One-way ANOVA | Group Effect | F (3, 16) = 12.90 | p=0.0002 | Control vs. CUMS | p=0.0002 |
|  | | |  |  |  |  | CUMS vs. CUMS+EA | p=0.0064 |
|  | | |  |  |  |  | CUMS vs. CUMS+sham | p=0.8300 |
| Fig.  3H | | | Two-way ANOVA | Type **×**Treatment | F (9, 36) = 3.560 | p=0.0030 | Control vs. CUMS | p=0.044 |
|  | | |  | Type | F (2.190, 26.28) = 27.29 | p<0.0001 | CUMS vs. CUMS+EA | p=0.0410 |
|  | | |  | Treatment | F (3, 12) = 47.70 | p<0.0001 | CUMS vs. CUMS+sham | p=0.0780 |

| Figure | Variable Pair | Method | n | Pearson's r | 95% CI | p-value |
| --- | --- | --- | --- | --- | --- | --- |
| Fig.  3I | Class I vs. DCX^+^ cells | Pearson's correlation coefficient | 24 | 0.8012 | 0.7696 to 0.9540 | p<0.0001 |
| Fig.  3J | Class I vs. Sucrose Preference | Pearson's correlation coefficient | 24 | 0.7466 | 0.7071 to 0.9399 | p<0.0001 |

Table S4. The statistical methods and data in Figure 4

| **Figure** | **Statistical Test** | | | **Main Effect / Interaction** | **F value (df)** | **p value** | **Post-hoc Comparison** | **Post-hoc p value** |  |
| --- | --- | --- | --- | --- | --- | --- | --- | --- | --- |
| Fig. 4B | One-way ANOVA | | | Group Effect | F (3, 16) = 7.484 | p=0.0024 | Control vs. CUMS | p=0.0093 |  |
|  |  | | |  |  |  | CUMS vs. CUMS+EA | p=0.0148 |  |
|  |  | | |  |  |  | CUMS vs. CUMS+sham | p=0.9998 |  |
| Fig. 4C | One-way ANOVA | | | Group Effect | F (3, 16) = 37.58 | p<0.0001 | Control vs. CUMS | p<0.0001 |  |
|  |  | | |  |  |  | CUMS vs. CUMS+EA | p<0.0001 |  |
|  |  | | |  |  |  | CUMS vs. CUMS+sham | p=0.9684 |  |
| Fig. 4D | Two-way ANOVA | | | Time x Column Factor | F (36, 48) = 25.29 | p<0.0001 |  |  |  |
|  |  | | | Time | F (3.746, 14.98) = 194.4 | p<0.0001 |  |  |  |
|  | | | |  | Column Factor | F (3, 16) = 21.39 | p=0.0063 |  |  |
| Fig. 4E | | | One-way ANOVA | | Group Effect | F (3, 16) = 18.12 | p<0.0001 | Control vs. CUMS | p=0.0328 |
|  | | | |  |  |  |  | CUMS vs. CUMS+EA | p<0.0001 |
|  | | | |  |  |  |  | CUMS vs. CUMS+sham | p=0.1195 |
| Figure | | | | Variable Pair | Method | n | Pearson's r | 95% CI | p-value |
| Fig. 4F | | | | Arg1^+^ microglia vs. DCX^+^ cells | Pearson's correlation coefficient | 20 | 0.2578 | 0.08413 to 0.7759 | p=0.0223 |
| Fig. 4G | | | | Arg1^+^ microglia vs. SPT | Pearson's correlation coefficient | 20 | 0.6111 | 0.5186 to 0.9096 | p<0.0001 |
| Fig. 4H-1 | | | | One-way ANOVA | Group Effect | F (3, 16) = 16.71 | p<0.0001 | Control vs. CUMS | p=0.0011 |
|  | | | |  |  |  |  | CUMS vs. CUMS+EA | p<0.0001 |
|  | | | |  |  |  |  | CUMS vs. CUMS+sham | p=0.6087 |
| Fig. 4H-2 | | | | One-way ANOVA | Group Effect | F (3, 16) = 22.28 | p<0.0001 | Control vs. CUMS | p=0.0001 |
|  | | | |  |  |  |  | CUMS vs. CUMS+EA | p<0.0001 |
|  | | | |  |  |  |  | CUMS vs. CUMS+sham | p=0.4222 |
| Fig. 4H-3 | | | | One-way ANOVA | Group Effect | F (3, 16) = 18.03 | p<0.0001 | Control vs. CUMS | p=0.0001 |
|  | | | |  |  |  |  | CUMS vs. CUMS+EA | p=0.0001 |
|  | | | |  |  |  |  | CUMS vs. CUMS+sham | p=0.8439 |
| Fig. 4G-4 | | | | One-way ANOVA | Group Effect | F (3, 16) = 33.41 | p<0.0001 | Control vs. CUMS | p<0.0001 |
|  | | | |  |  |  |  | CUMS vs. CUMS+EA | p<0.0001 |
|  | | | |  |  |  |  | CUMS vs. CUMS+sham | p<0.0001 |

Table S5. The statistical methods and data in Figure 5

| **Figure** | **Statistical Test** | | | **Main Effect / Interaction** | **F value (df)** | **p value** | **Post-hoc Comparison** | **Post-hoc p value** |  |
| --- | --- | --- | --- | --- | --- | --- | --- | --- | --- |
| Fig. 5D | One-way ANOVA | | | Group Effect | F (3, 20) = 31.18 | p<0.0001 | Control vs. CUMS | p<0.001 |  |
|  |  | | |  |  |  | CUMS vs. CUMS+EA | p<0.001 |  |
|  |  | | |  |  |  | CUMS vs. CUMS+PLX+EA | p<0.001 |  |
| Fig. 5E | One-way ANOVA | | | Group Effect | F (3, 20) = 8.814 | p=0.0006 | Control vs. CUMS | p=0.0153 |  |
|  |  | | |  |  |  | CUMS vs. CUMS+EA | p=0.0005 |  |
|  | | | |  |  |  |  | CUMS vs. CUMS+PLX+EA | p=0.0209 |
| Fig. 5F | | | One-way ANOVA | | Group Effect | F (3, 16) = 15.28 | p<0.0001 | Control vs. CUMS | p=0.0009 |
|  | | | |  |  |  |  | CUMS vs. CUMS+EA | p=0.0042 |
|  | | | |  |  |  |  | CUMS vs. CUMS+PLX+EA | p=0.0014 |
| Fig. 5G | | | | One-way ANOVA | Group Effect | F (3, 28) = 45.56 | p<0.0001 | Control vs. CUMS | p<0.0001 |
|  | | | |  |  |  |  | CUMS vs. CUMS+EA | p<0.0001 |
|  | | | |  |  |  |  | CUMS vs. CUMS+PLX+EA | p<0.0001 |
| Fig. 5H | | | | One-way ANOVA | Group Effect | F (3, 28) = 13.58 | p<0.0001 | Control vs. CUMS | p=0.0004 |
|  | | | |  |  |  |  | CUMS vs. CUMS+EA | p=0.0015 |
|  | | | |  |  |  |  | CUMS vs. CUMS+PLX+EA | p=0.0009 |
| Fig. 5I-1 | | | | One-way ANOVA | Group Effect | F (3, 28) = 24.62 | p<0.0001 | Control vs. CUMS | p=0.0017 |
|  | | | |  |  |  |  | CUMS vs. CUMS+EA | p=0.0104 |
|  | | | |  |  |  |  | CUMS vs. CUMS+PLX+EA | p<0.0001 |
| Fig. 5I-2 | | | | One-way ANOVA | Group Effect | F (3, 28) = 26.60 | p<0.0001 | Control vs. CUMS | p<0.0001 |
|  | | | |  |  |  |  | CUMS vs. CUMS+EA | p<0.0001 |
|  | | | |  |  |  |  | CUMS vs. CUMS+PLX+EA | p<0.0001 |

Table S6. The statistical methods and data in Figure 6

| **Figure** | **Statistical Test** | | | **Main Effect / Interaction** | **F value (df)** | **p value** | **Post-hoc Comparison** | **Post-hoc p value** |  |
| --- | --- | --- | --- | --- | --- | --- | --- | --- | --- |
| Fig. 6B | One-way ANOVA | | | Group Effect | F (3, 20) = 28.44 | p<0.0001 | Control vs. CUMS | p=0.0023 |  |
|  |  | | |  |  |  | CUMS vs. CUMS+EA | p<0.0001 |  |
|  |  | | |  |  |  | CUMS vs. CUMS+PLX+EA | p=0.0001 |  |
| Fig. 6C | One-way ANOVA | | | Group Effect | F (3, 29) = 28.14 | p=0.0006 | Control vs. CUMS | p=0.0017 |  |
|  |  | | |  |  |  | CUMS vs. CUMS+EA | p<0.0001 |  |
|  | | | |  |  |  |  | CUMS vs. CUMS+PLX+EA | p<0.0001 |
| Fig. 6D | | | One-way ANOVA | | Group Effect | F (3, 32) = 25.60 | p<0.0001 | Control vs. CUMS | p=0.0002 |
|  | | | |  |  |  |  | CUMS vs. CUMS+EA | p<0.0001 |
|  | | | |  |  |  |  | CUMS vs. CUMS+PLX+EA | p<0.0001 |
| Fig.  6F | | | | One-way ANOVA | Group Effect | F (3, 20) = 11.54 | p=0.0001 | Control vs. CUMS | p=0.0004 |
|  | | | |  |  |  |  | CUMS vs. CUMS+EA | p=0.0005 |
|  | | | |  |  |  |  | CUMS vs. CUMS+PLX+EA | p=0.0445 |
| Fig.  6G | | | | One-way ANOVA | Group Effect | F (3, 20) = 6.049 | P=0.0004 | Control vs. CUMS | p=0.0331 |
|  | | | |  |  |  |  | CUMS vs. CUMS+EA | p=0.0213 |
|  | | | |  |  |  |  | CUMS vs. CUMS+PLX+EA | p=0.0320 |
| Fig.  6H | | | | One-way ANOVA | Group Effect | F (3, 20) = 16.21 | p<0.0001 | Control vs. CUMS | p=0.0003 |
|  | | | |  |  |  |  | CUMS vs. CUMS+EA | p=0.0124 |
|  | | | |  |  |  |  | CUMS vs. CUMS+PLX+EA | p=0.0018 |
| Fig.  6I | | | | One-way ANOVA | Group Effect | F (3, 20) = 13.15 | p<0.0001 | Control vs. CUMS | p=0.0004 |
|  | | | |  |  |  |  | CUMS vs. CUMS+EA | p=0.0053 |
|  | | | |  |  |  |  | CUMS vs. CUMS+PLX+EA | p=0.0060 |
| Fig.  6J-1 | | | | One-way ANOVA | Group Effect | F (3, 20) = 8.858 | p=0.0006 | Control vs. CUMS | p=0.0075 |
|  | | | |  |  |  |  | CUMS vs. CUMS+EA | p=0.0092 |
|  | | | |  |  |  |  | CUMS vs. CUMS+PLX+EA | p=0.0087 |
| Fig.  6J-2 | | | | One-way ANOVA | Group Effect | F (3, 20) = 0.3038 | p=0.8223 | Control vs. CUMS | p=0.8960 |
|  | | | |  |  |  |  | CUMS vs. CUMS+EA | p=0.8283 |
|  | | | |  |  |  |  | CUMS vs. CUMS+PLX+EA | p=0.9996 |
| Fig.  6J-3 | | | | One-way ANOVA | Group Effect | F (3, 20) = 10.39 | p=0.0002 | Control vs. CUMS | p=0.0034 |
|  | | | |  |  |  |  | CUMS vs. CUMS+EA | p=0.0124 |
|  | | | |  |  |  |  | CUMS vs. CUMS+PLX+EA | p=0.0055 |

| Figure | Variable Pair | Method | n | Pearson's r | 95% CI | p-value |
| --- | --- | --- | --- | --- | --- | --- |
| Fig. 6K | PKA protein level vs. MECP2 protein level | Pearson's correlation coefficient | 24 | -0.4337 | -0.7125 to -0.03677 | p=0.0342 |

| Fig. 6L-1 | Unpaired t test | t=4.774, df=6 | Ctrl vs H89 | p=0.0031 |
| --- | --- | --- | --- | --- |
| Fig. 6L-2 | Unpaired t test | t=2.497, df=6 | Ctrl vs H89 | p=0.0467 |

Table S7. The statistical methods and data in Figure 7

| **Figure** | **Statistical Test** | | | **Main Effect / Interaction** | **F value (df)** | **p value** | **Post-hoc Comparison** | **Post-hoc p value** |  |
| --- | --- | --- | --- | --- | --- | --- | --- | --- | --- |
| Fig. 7D | One-way ANOVA | | | Group Effect | F (3, 28) = 19.33 | p<0.0001 | Control vs. CUMS | p<0.0001 |  |
|  |  | | |  |  |  | CUMS vs. CUMS+EA | p=0.0010 |  |
|  |  | | |  |  |  | CUMS vs. CUMS+EA+ANA-12 | p=0.0430 |  |
| Fig. 7E | One-way ANOVA | | | Group Effect | F (3, 12) = 22.63 | p=0.0006 | Control vs. CUMS | p<0.0001 |  |
|  |  | | |  |  |  | CUMS vs. CUMS+EA | p=0.0018 |  |
|  | | | |  |  |  |  | CUMS vs. CUMS+EA+ANA-12 | p=0.0032 |
| Fig. 7F | | | One-way ANOVA | | Group Effect | F (3, 12) = 79.36 | p<0.0001 | Control vs. CUMS | p<0.0001 |
|  | | | |  |  |  |  | CUMS vs. CUMS+EA | p<0.0001 |
|  | | | |  |  |  |  | CUMS vs. CUMS+EA+ANA-12 | p<0.0001 |
| Fig. 7G | | | | One-way ANOVA | Group Effect | F (3, 12) = 12.38 | p=0.0006 | Control vs. CUMS | p=0.0015 |
|  | | | |  |  |  |  | CUMS vs. CUMS+EA | p=0.0367 |
|  | | | |  |  |  |  | CUMS vs. CUMS+EA+ANA-12 | p=0.0347 |
| Fig. 7H | | | | One-way ANOVA | Group Effect | F (3, 12) = 12.40 | P=0.0005 | Control vs. CUMS | p=0.0055 |
|  | | | |  |  |  |  | CUMS vs. CUMS+EA | p=0.0339 |
|  | | | |  |  |  |  | CUMS vs. CUMS+EA+ANA-12 | p=0.0064 |

Table S8. The statistical methods and data in Figure 8

| **Figure** | **Statistical Test** | | | **Main Effect / Interaction** | **F value (df)** | **p value** | **Post-hoc Comparison** | **Post-hoc p value** |  |
| --- | --- | --- | --- | --- | --- | --- | --- | --- | --- |
| Fig. 8B | One-way ANOVA | | | Group Effect | F (3, 28) = 26.86 | p<0.0001 | Control vs. CUMS | p<0.0001 |  |
|  |  | | |  |  |  | CUMS vs. CUMS+EA | p=0.0028 |  |
|  |  | | |  |  |  | CUMS vs. CUMS+EA+ANA-12 | p=0.0013 |  |
| Fig. 8C | One-way ANOVA | | | Group Effect | F (3, 28) = 19.33 | p<0.0001 | Control vs. CUMS | p<0.0001 |  |
|  |  | | |  |  |  | CUMS vs. CUMS+EA | p=0.0010 |  |
|  | | | |  |  |  |  | CUMS vs. CUMS+EA+ANA-12 | p=0.0034 |
| Fig. 8D-1 | | | One-way ANOVA | | Group Effect | F (3, 28) = 17.85 | p<0.0001 | Control vs. CUMS | p=0.0061 |
|  | | | |  |  |  |  | CUMS vs. CUMS+EA | p=0.0013 |
|  | | | |  |  |  |  | CUMS vs. CUMS+EA+ANA-12 | p<0.0001 |
| Fig. 8D-2 | | | | One-way ANOVA | Group Effect | F (3, 28) = 22.03 | p<0.0001 | Control vs. CUMS | p<0.0001 |
|  | | | |  |  |  |  | CUMS vs. CUMS+EA | p<0.0001 |
|  | | | |  |  |  |  | CUMS vs. CUMS+EA+ANA-12 | p=0.0007 |
| Fig. 8F | | | | One-way ANOVA | Group Effect | F (3, 28) = 7.871 | P=0.0006 | Control vs. CUMS | p=0.0041 |
|  | | | |  |  |  |  | CUMS vs. CUMS+EA | p=0.0481 |
|  | | | |  |  |  |  | CUMS vs. CUMS+EA+ANA-12 | p=0.0311 |
| Fig. 8H | | | | One-way ANOVA | Group Effect | F (3, 28) = 14.69 | p<0.0001 | Control vs. CUMS | p=0.0178 |
|  | | | |  |  |  |  | CUMS vs. CUMS+EA | p<0.0001 |
|  | | | |  |  |  |  | CUMS vs. CUMS+EA+ANA-12 | p<0.0001 |
