# Supplementary material for: Electroacupuncture promotes BDNF-dependent neurogenesis via microglial reprogramming in a chronic stress model
Source: Chin Med. 2026 Feb 3;21:62. doi: 10.1186/s13020-026-01334-y (PMC12866076; doi:10.1186/s13020-026-01334-y)
Supplement: Supplementary file 6 — Supplementary Material 6. Table S9. PCR primers [file 13020_2026_1334_MOESM6_ESM.docx]

Methods

Table S9. PCR primers

| Gene names | primers |
| --- | --- |
| *IL6* | Forward: 5’- GCCTTCTTGGGACTGATGCT-3’  Reverse: 5’- GCCACTCCTTCTGTGACTCC-3’ |
| *TNFα* | Forward: 5'- AGGCACTCCCCCAAAAGATG-3’  Reverse: 5’-TGGTGGTTTGTGAGTGTGAGG-3’ |
| *IL4* | Forward: 5’-TCTCGAATGTACCAGGAGCCATATC-3’  Reverse: 5’-AGCACCTTGGAAGCCCTACAGA-3’ |
| *TGFβ* | Forward: 5’-GACCGCAACAACGCCATCTA-3’  Reverse: 5’-GGCGTATCAGTGGGGGTCAG-3’ |
| *CREB* | Forward: 5'- CATCTGCTCCCACTGTAACCT-3’  Reverse: 5'- CTTCAATCCTTGGCACCCCT-3’ |
| *TRKB* | Forward: 5’- AAGTTTTCCTTGCCGAGTGC-3’  Reverse: 5’- CCTCCACACAGACACCGTAG-3’ |
| *BDNF* | Forward: 5’-GAGCTGAGCGTGTGTGACAG-3’  Reverse: 5’-CGCCAGCCAATTCTCTTTTTGC-3’ |
| *β-actin* | Forward: 5'-CCGTGAAAAGATGACCCAGATC-3’  Reverse: 5'-CACAGCCTGGATGGCTACGT-3’ |
