# Supplementary material for: Electroacupuncture promotes BDNF-dependent neurogenesis via microglial reprogramming in a chronic stress model
Source: Chin Med. 2026 Feb 3;21:62. doi: 10.1186/s13020-026-01334-y (PMC12866076; doi:10.1186/s13020-026-01334-y)
Supplement: Supplementary file 7 — Supplementary Material 7. Table S10. List of primary and secondary antibodies [file 13020_2026_1334_MOESM7_ESM.docx]

Methods

Table S10. List of primary and secondary antibodies

| REAGENT or RESOURCE | SOURCE | IDENTIFIER |
| --- | --- | --- |
| Immunofluorescence Antibody |  |  |
| Goat polyclonal anti-Iba1; 1:800 | Wako | Cat#019-19741 |
| Rabbit monoclonal anti-CD68; 1:800 | HUABIO | Cat# PSH05-47 |
| Rabbit monoclonal antibody-GFAP; 1:500 | HUABIO | Cat# ET1601-23 |
| Rabbit monoclonal antibody-SOX2; 1:800 | HUABIO | Cat# HA721155 |
| Rabbit monoclonal antibody-Arg1 | HUABIO | Cat# PSH09-78 |
| Rabbit monoclonal antibody-NeuN;1：800 | HUABIO | Cat# SR45-07 |
| Guinea pig monoclonal anti-DCX; 1:500 | Oasis Biofarm | Cat# OB-PGP019 |
| Rat monoclonal antibody-BDNF; 1:500 | Oasis Biofarm | Cat# OB-PRT178-02 |
| AF488 donkey anti-goat; 1:1,000 | Jackson Immuno | Cat#: 705-545-003 |
| AF568 donkey anti-goat; 1:1,000 | Thermo Fisher | Cat#: A11057 |
| AF568 donkey anti-rabbit; 1:1,000 | Thermo Fisher | Cat#: A10042 |
| AF594 donkey anti-rat; 1:1,000 | Oasis Biofarm | Cat#: D-RT594 |
| AF488 donkey anti-rabbit; 1:1,000 | Oasis Biofarm | Cat#: D-RB488 |
| AF594 donkey anti-Guinea pig; 1:1,000 | Oasis Biofarm | Cat#: D-GP594 |
| AF488 donkey anti-Guinea pig; 1:1,000 | Oasis Biofarm | Cat#: D-GP488 |
| WB antibody |  |  |
| CREB-1 (PT0516R) PT™ Rabbit mAb; 1:2000 | Immunoway | Cat#YM8342 |
| β-actin Rabbit mAb; 1:20000 | Cell Signaling Technology | Cat#4967 |
| Doublecortin (PT0871R) PT™ Rabbit mAb; 1:10000 | Immunoway | Cat#YM8640 |
| MECP2 (PT0726R) PT™ Rabbit mAb; 1:10000 | Immunoway | Cat#YM8090 |
| BDNF (PT0858R) PT™ Rabbit mAb;1;10000 | Immunoway | Cat#YM8627 |
| Trk B Rabbit pAb; 1:2000 | Immunoway | Cat#YT4743 |
| Trk B (Phospho Tyr706) Rabbit pAb;1:2000 | Immunoway | Cat#YP0270 |
| PKAα/β Rabbit pAb; 1:2000 | Immunoway | Cat#YT3748 |
| Goat Anti Rabbit IgG(H+L) (HRP);1:2000 | Immunoway | Cat#RS0002 |
